# Multi-Statistical Pragmatic Framework to Study UV Exposure Effects via VIS Reflectance in Automotive Polymer Components

**DOI:** 10.3390/polym17212849

**Published:** 2025-10-25

**Authors:** Jose Amilcar Rizzo-Sierra, Luis Alvaro Montoya-Santiyanes, Cesar Isaza, Karina Anaya, Cristian Felipe Ramirez-Gutierrez, Jonny Paul Zavala de Paz

**Affiliations:** 1 Cuerpo Académico de Tecnologías de la Información y Comunicación Aplicada, Universidad Politécnica de Querétaro (UPQ), Carretera Estatal 420 SN, El Marqués, Santiago de Querétaro 76240, Mexico; cesar.isaza@upq.edu.mx (C.I.); karina.anaya@upq.mx (K.A.); cristian.ramirez@upq.edu.mx (C.F.R.-G.); 2 Unidad de Alta Tecnología, Facultad de Ingeniería, Universidad Nacional Autónoma de México (UNAM), Fray Antonio de Monroy e Hijar 260, Villas del Mesón, Juriquilla, Santiago de Querétaro 76230, Mexico; montoyas@unam.edu

**Keywords:** automotive polymers, UV degradation, reflectance spectroscopy, statistical analyses

## Abstract

This study evaluates the cosmetic degradation of polyethylene (PE) and polypropylene (PP) automotive components under four exposure scenarios—no exposure, outdoor exposure with and without glass shielding, and accelerated UV chamber weathering (ASTM G154)—through the evolution of visible (VIS) reflectance. Thirty-two samples (16 PE, 16 PP) were monitored over five time points; surface reflectance was recorded at 21 wavelengths and summarized into seven VIS bands, and hardness (Shore D) was measured pre/post-exposure. Repeated-measures univariate and multivariate analyses consistently revealed significant effects of Condition, Time, and their interaction on reflectance, with initial-reflectance adjustment improving inference stability across bands. PE exhibited more gradual and coherent reflectance decay, whereas PP showed greater band-to-band variability—most notably under UV chamber exposure. Additionally, hardness decreased in most exposed groups, aligning with optical changes. To place spectral trajectories in a kinetic context, a family of exponential models with small-sample information criterion selection was fitted, yielding η(t)—a dimensionless degradation efficiency summarizing spectral change. The contribution of this work is a multi-statistical framework—combining VIS-band-aware summaries with covariate-adjusted univariate/multivariate testing—that supports comparisons across materials and exposure conditions, underscoring the practical value of UV chamber protocols as surrogates for outdoor weathering. In sum, the study demonstrates the effectiveness of multivariate and covariate-adjusted models in quantifying optical degradation of polyolefins, offering pragmatic guidance for assessing mid- to long-term performance in automotive applications.

## 1. Introduction

Polyolefins, such as polyethylene (PE) and polypropylene (PP), i.e., thermoplastic polymers, are extensively utilized in automotive applications due to their favorable mechanical properties, cost-effectiveness, and ease of processing [1,2]. However, their susceptibility to environmental degradation, particularly under ultraviolet (UV) radiation, poses significant challenges for long-term durability in terms of mechanical performance and aesthetic/cosmetic qualities [3,4]. Degradation processes such as thermal aging and mechanical wear can significantly alter their physical and chemical properties over time [5,6,7]. In particular, exposure to UV light initiates photo-oxidative reactions in these polymers, leading to chain scission, cross-linking, and the formation of carbonyl groups, which collectively result in discoloration/cosmetic degradation, embrittlement, and loss of mechanical integrity [8,9,10,11,12]. These last subjects being critical for the automotive industry.

The automotive industry often employs accelerated aging tests, such as UV exposure chambers, in order to predict the service life of polymeric components [13,14,15,16,17]. These tests simulate prolonged environmental exposure within a condensed timeframe, allowing for the assessment of material performance under controlled conditions [16,18,19,20]. Standards such as ASTM D5208-14 and ISO 4892-3:2024 provide guidelines for conducting such evaluations [21,22]. Additionally, real-world outdoor exposures, both with and without protective barriers like glass, provide insights into the environmental durability of these materials [23,24,25,26].

On the other hand, reflectance spectroscopy in the visible range, employed in the present study, serves as a non-destructive method to monitor the surface degradation of polymers. For example, Goddin et al. reviewed the effectiveness of VIS–SWIR (visible to short-wave infrared) spectroscopy in distinguishing plastic litter on and below water surfaces. It supports the method’s ability to non-invasively monitor polymer presence and condition, reinforcing its utility in environmental diagnostics through spectral reflectance analysis [27]. Goustoridis et al. [28] showcase white light reflectance spectroscopy (WLRS) as a rapid, accurate, and contactless tool for determining the thickness and transformation of films and layers, including polymer coatings. It highlights WLRS’s versatility in tracking real-time surface changes like dissolution or degradation, without damaging the sample. Makki et al. [29] employed reflectance spectroscopy to quantify weathering-induced changes in polymer films. The technique proved effective in identifying surface color shifts and chemical transformations due to UV exposure, demonstrating its reliability for non-destructive monitoring. Chaudhary et al. [30] reported on using UV-VIS reflectance analysis to examine polymer degradation. The method was essential for identifying shifts in optical properties related to molecular structural changes, underscoring its role in non-destructive diagnostics. Matuana et al. [31] used reflectance spectroscopy to assess UV-induced surface degradation in wood–plastic composites. Changes in reflectance spectra, especially within the visible range, were directly linked to color fading and chemical deterioration. Choi et al. [32] investigated whitening in UV-exposed elastomers, and linked surface degradation to spectral variations in the visible region. Reflectance data allowed for clear, non-destructive identification of photo-induced chemical changes on the material surface. Exposito et al. [33] detail how UV-VIS spectroscopy detects photodegradation by monitoring new absorption bands and reflectance losses. It is especially useful for mapping oxidative changes and morphological deterioration over time. Wijdekop et al. [34] applied reflectance spectroscopy to PVC-coated steel undergoing weathering. The method successfully captured gradual discoloration due to UV and environmental stress, showing strong potential for long-term, non-invasive surface quality evaluation. Gulmine et al. [35] tracked the chemical breakdown and surface oxidation of polyethylene during UV aging. Variations in spectral intensity served as indicators of both physical and chemical deterioration. Komiya et al. assessed cosmetic degradation and microstructural alterations via reflectance data in polymers, with clear visibility of changes in the VIS spectrum due to photo-oxidation and cross-linking reactions [36].

Recent work has also emphasized how polymer formulation—such as resin density, stabilizer dosage, pigment loading, and incorporation of nanoparticles—directly affects the rate and type of UV-induced degradation. For example, Du et al. [21] quantitatively analyzed LDPE sheets with varying thicknesses and pro-oxidant metal stearate contents, revealing significant effects on carbonyl index, molecular weight reduction, and VIS reflectance shifts under ASTM UV weathering. Zhang et al. [37] analyzed polyethylene samples with differing densities and nanoparticle fillers, establishing correlation between formulation parameters and surface degradation kinetics assessed via reflectance-based techniques. Andrady et al. [38] investigated LDPE laminates exposed to UV radiation in both air and seawater. Their quantitative FTIR depth-profiling and tensile property assessment revealed diffusion-controlled photo-oxidation localized in a thin surface layer and slower degradation kinetics in seawater due to limited oxygen solubility. Weizman et al. [39] quantitatively demonstrated that incorporating nanoscale UV stabilizers into LDPE films significantly affects photodegradation kinetics. Using UV-VIS reflectance and FTIR analysis, they showed that nanoparticle-enhanced formulations reduce stabilizer loss and delay surface oxidation, emphasizing the importance of additive design in modulating optical aging under UV exposure. Brostow et al. [40] systematically studied polypropylene films containing HALS and nano-ZnO under accelerated outdoor UV exposure, demonstrating that combinations of stabilizers confer a measurable increase in UV resistance and tensile strength retention. Cavalcanti & Rabello [41] evaluated polypropylene films with different levels of red pigment and UV-stabilizers (UV absorbers and HALS), showing that pigment concentration quantitatively alters color shift ($\Delta E^{*}$) and mechanical degradation trajectories. Grigoriadou et al. [42] found that HDPE nanocomposites containing SiO_2_ or montmorillonite nanoparticles catalyze UV degradation, resulting in increased carbonyl and vinyl groups, surface cracking, and altered optical properties detectable via spectroscopy. Regarding other recent polymer-based industrial applications, such as CNT/epoxy resins with PCL as fillers, Xian et al. also quantitatively link formulation/preparation parameters to design-driven performance metrics [43], validating the perspective applied here to the rate and mode of UV-driven degradation in formulated polyolefins intended for outdoor use.

This range of quantitative findings directly informs the experimental design contemplated in this study, excepting the pigment/filler/stabilizer composition factor, outside of its scope. By systematically varying polymer type (PE vs. PP) and exposure modes (outdoor vs. artificial UV), we aim to investigate their combined influence on VIS reflectance degradation trajectories. This integration of formulation technology and controlled experimental design helps to ensure a rigorous quantitative comparison of cosmetic stability in automotive polymer components. In addition, particularly regarding reflectance–transmittance data gathering and analysis, these previous works indicate that changes in polymer spectra can signal alterations in surface chemistry and morphology due to weathering/cosmetic degradation [44,45,46]. Specifically, the emergence of new absorption bands or shifts in existing ones can be correlated with the formation of degradation products or sub-products such as carbonyl compounds [47,48,49,50]. In summary, characterizing and understanding the degradation behavior of polymers such as PE and PP is crucial for predicting their service life and ensuring reliability and cosmetic standards compliance for automotive industry components.

In this study, we investigate the degradation behavior of eight automotive plastic parts—four made of PE and four of PP—subjected to four distinct environmental exposure conditions: (C1) no exposure (control), (C2) outdoor exposure with glass protection, (C3) outdoor exposure without protection, and (C4) accelerated aging via UV chamber simulation. In particular, ASTM G154 protocol ([51]) was implemented. Reflectance measurements were conducted at five time points (T0 to T4) across 21 wavelengths grouped into seven VIS-bands (Violet to Red), with each band comprising three closely spaced wavelengths in order to serve as statistical replicates. The experimental design incorporates both between-subjects factors (Material and Exposure Condition) and a within-subjects factor (Time). Given the repeated measures and the hierarchical structure of the data, statistical analyses were performed using repeated-measures ANOVA and ANCOVA for univariate responses and MANOVA and MANCOVA for multivariate responses. Initial reflectance values at T0 were included as covariates to adjust for baseline differences between samples. This comprehensive approach aims to consider the effects of material type, exposure environmental condition, and time on the optical properties via VIS reflectance of automotive polymers (PP and PE) over time, providing a framework with valuable insights for the development of more durable and cosmetically stable materials in automotive applications.

## 2. Materials and Methods

### 2.1. Samples and Experimental Design

The study was conducted using 8 distinct automotive plastic part samples, labeled as subjects, from Subject 1 to 8. The first four (Subject 1 to Subject 4) were made of polyethylene (PE), while the remaining four (Subject 5 to Subject 8) were made of polypropylene (PP). Each of these subjects was exposed to one of four distinct environmental exposure conditions:

C1: No exposure (control).
C2: Outdoor environmental exposure with glass protection.
C3: Outdoor environmental exposure without glass protection.
C4: Accelerated aging via UV chamber simulation (ASTM G154 protocol [51]).

Reflectance was measured for each subject at five time points:

- **T0:** Initial time (before exposure).
- **T1–T4:** After 1/4, 2/4, 3/4, and 4/4 of the experiment duration, respectively. In this particular respect, the experimentation run extended for a period of 4 months. A uniform temporal spacing was selected to ensure consistent tracking of mid- to long-term cumulative environmental exposure under outdoor and artificial weathering/aging scenarios for the type of materials considered, as supported by studies such as ref. [52] regarding mechanical degradation, and ref. [53] on UV aging. While front-loaded sampling designs may be preferable when early degradation dynamics are dominant, the mentioned literature precedents indicate that reflectance variation is gradual, justifying neither denser early sampling nor reflectance rate-of-change analyses in this study. On the other hand, fixed-interval data were required to develop repeated-measures statistical modeling frameworks. Tests were conducted on the campus of *Universidad Politécnica de Querétaro* (20°32′48″ N, 100°16′28″ W), *El Marqués, Querétaro, México*. The time lapse went from July to October 2024, during the local rainy season. Climatological data from Querétaro Intercontinental Airport indicate that, over these four months, daily mean temperatures typically ranged from 14 °C (overnight lows) to 25 °C (daytime highs), with relative humidity between 64% and 69%. Monthly accumulated precipitation was approximately 96 mm in July, 86 mm in August, 87 mm in September, and 30 mm in October [54].

**Reflectance measurements** were taken in the visible light spectrum using 21 specific wavelengths grouped into 7 VIS-bands, each composed of 3 closely spaced wavelengths:

- **Violet:** 400, 410, 420 [nm].
- **Blue:** 450, 460, 470 [nm].
- **Cyan:** 480, 490, 500 [nm].
- **Green:** 520, 530, 540 [nm].
- **Yellow:** 560, 570, 580 [nm].
- **Orange:** 600, 610, 620 [nm].
- **Red:** 640, 650, 660 [nm].

That meant that each wavelength within a band was treated as a replicate for statistical purposes. The Subject ID and material are not independent, as the type of material is systematically associated with specific subject IDs.

**Shore D Hardness Measurements:** Surface hardness was evaluated at two stages (T0 and T4) using the Shore D scale, a standardized metric for assessing the rigidity of polymers. For each material and exposure condition, measurements were obtained from four independent samples (subjects), each tested once per stage. This allowed quantification of mechanical degradation due to UV exposure. The main features of the Shore D digital durometer used are present in Table 1.

**Reflectance Measurements:** Reflectances were realized with a Konica Minolta CM-700d spectrophotometer, whose main features are described in Table 2.

### 2.2. Statistical Structure of the Experiment

A structured experimental design with both between-subject and within-subject factors was employed. The statistical analysis framework rests upon clear identification and distinction between the following:

- **Independent variables (factors):** Variables manipulated or categorized in the experiment to assess their influence on the outcomes.
- **Dependent variables (responses):** Quantities that are measured as outcomes and are hypothesized to be affected by the independent variables.
- **Between-subjects factors:** Factors for which each subject (i.e., each individual subject) is observed under only one level. These define groups of subjects.
- **Within-subjects factors (repeated measures):** Factors for which each subject is observed at multiple levels. These correspond to repeated measurements over time or under different internal conditions.

The experimental structure employed can be described as follows:

#### 2.2.1. Dependent (Response) Variables

- **Univariate case:** Mean reflectance per VIS-band (7 VIS-bands total), computed by averaging the reflectance over the 3 closely spaced wavelengths within each band.
- **Multivariate case:** The 7-dimensional reflectance vector per observation, with each component corresponding to the mean reflectance in one VIS-band (Violet to Red).

#### 2.2.2. Independent Variables (Factors)

- **Between-subject factors:**
  − **Material (2 levels):** Polyethylene (PE), polypropylene (PP). Note: Material is not independent from Subject ID, as each subject is uniquely assigned a material type.
  − **Condition of Exposure (4 levels):** C1 (control), C2 (glass-protected), C3 (unprotected), C4 (UV chamber).
- **Within-subject factors:**
  − **Time (5 levels):** T0 (initial), T1, T2, T3, T4. All subjects were measured repeatedly at the same 5 time points.

#### 2.2.3. Covariates (for ANCOVA/MANCOVA)

- **Initial Reflectance (T0):** For each VIS-band (or the 7-dimensional vector), the reflectance at time T0 is used as a covariate. This adjustment allows the models to control for baseline variability among subjects that may arise due to inherent color differences, pigmentation, or surface features.

### 2.3. Experimental Units and Repetition

- Each of the 8 automotive part samples is treated as a subject.
- Each subject is assigned a unique combination of material and exposure condition.
- Reflectance is recorded for each subject at 5 time points and for 21 wavelengths grouped into 7 VIS-bands, with 3 replicate wavelengths per band.
- For statistical analysis, the mean reflectance across the 3 replicates within a band is treated as the response variable (univariate) or part of a vector of responses (multivariate).

This design allows the analysis of the following:

- Temporal evolution of reflectance (within-subject effects),
- Material and exposure-related differences (between-subject effects),
- Adjustments for initial reflectance variability (via covariates).

The repeated-measures design ensures that each subject serves as its own control over time, increasing the statistical power and sensitivity to changes in reflectance behavior.

### 2.4. Statistical Analyses

Regarding this present experiment, the following analyses were conducted using MATLAB R2024b and appropriate statistical packages. Custom scripts were developed specifically, and the structure of the analyses was guided by the nature of the reflectance data (longitudinal, repeated-measures, multiband), the presence of both between- and within-subject factors, and the interest in controlling for initial reflectance variability. The primary inferential route of the present study is a repeated-measures MANCOVA on the 7-band reflectance vector with T0 as covariate, testing Material, Condition, Time, and their interactions. When multivariate significance arises, band-wise ANCOVAs (with T0 as covariate) are used to localize effects and aid interpretation within the visible spectrum. In addition, RM-MANOVA and unadjusted ANOVA are retained and reported as well; as complementary, coarse-sensitivity benchmarks related to statistical analyses/models without baseline adjustment. Shore D hardness was analyzed via one-way/two-way ANOVA with appropriate post-hoc tests as a material performance endpoint. Finally, bounded exponential summaries η(t) (first-order, stretched/lag, constrained bi-exponential; AICc selection) were developed, enabling descriptive kinetics ($\eta_{inf}$, t1/2) that complement the inferential framework.

1. **Shore D Hardness Analysis**
  Mechanical degradation was assessed using Shore D hardness values obtained before (T0) and after (T4) exposure. Two complementary statistical approaches were employed:
  - *Paired Tests:* For each material and exposure condition, post-exposure hardness was compared to reference values using paired *t*-tests or Wilcoxon signed-rank tests, depending on the normality of the paired differences (tested via Lilliefors test).
  - *One-Way ANOVA with Tukey Post Hoc:* For each material separately, a one-way ANOVA was conducted to compare hardness values across the four exposure conditions. Tukey’s test was used for multiple comparisons.
  These tests were used to detect significant mechanical degradation due to environmental exposure and to compare the resilience of each polymer under the different aging scenarios.
2. **Material Non-Specific ANOVA and ANCOVA**
  Mean reflectance per VIS-band was used as the dependent variable. The effects of material, exposure condition, and time were evaluated using repeated-measures ANOVA and ANCOVA. The initial reflectance (T0) was included as a covariate in ANCOVA. For each of the 7 VIS-bands (Violet to Red), the mean reflectance (averaged over its 3 wavelengths) was analyzed independently using univariate repeated-measures ANOVA. The between-subjects factors were *Material* (PE vs. PP) and *Condition of Exposure* (C1–C4), and the within-subjects factor was *Time* (T0–T4). In ANCOVA models, the initial reflectance value at T0 for each band was included as a covariate to adjust for baseline differences.
3. **Material-Specific ANOVA and ANCOVA**
  For polyethylene and polypropylene materials, respectively, mean reflectance per VIS-band was analyzed with exposure condition and time as factors. Again, ANCOVA models included T0 reflectance as a covariate. The analyses in the previous item were repeated separately for the PE group and the PP group to explore material-specific effects of exposure condition and time. This allowed for better isolation of degradation behavior and interaction effects within each material class.
4. **Material-Non-specific MANOVA and MANCOVA**
  Identical structure to the previous item, to confirm reproducibility and consistency under alternative implementations or assumptions. The reflectance data were aggregated into 7-dimensional vectors (one per VIS-band) per subject per time point. These vectors were used as multivariate responses in a repeated-measures MANOVA framework, with *Material* and *Condition* as between-subject factors and *Time* as the within-subject factor. In MANCOVA, the corresponding 7-dimensional vector at T0 was used as a covariate to remove initial heterogeneity.
5. **Material-Specific MANOVA and MANCOVA**
  For each material type, multivariate reflectance responses (7 bands) were analyzed as a function of exposure condition and time, with or without the corresponding initial reflectance vector as covariate. The multivariate analysis approach was applied separately to PE and PP groups, allowing the multivariate trajectory of degradation across the 7 VIS-bands to be explored as a function of exposure condition and time. MANCOVA models again included initial (T0) reflectance vectors as covariates.

The statistical workflow was designed to evaluate how visible reflectance evolves across environmental conditions, sampling times, and material types while accounting for initial differences between samples. All analyses were conducted in MATLAB R2024b using both univariate and multivariate approaches. On the other hand, prior to hypothesis testing, the global normality of the reflectance and hardness data was confirmed by Shapiro–Wilk tests on each group defined by Material, Condition, and Time (p>0.05 in all cases).

For the Shore D hardness data, paired comparisons against the reference condition were evaluated by first testing the differences for normality using the Lilliefors test (MATLAB’s lillietest). When normality was not rejected, paired *t*-tests were applied; otherwise, Wilcoxon signed–rank tests were used. Homogeneity of variances across conditions was assessed by Levene’s test (vartestn with ‘LeveneAbsolute’, α=0.05). One-way ANOVA models were then fitted separately for polyethylene and polypropylene, followed by Tukey–Kramer multiple comparisons (multcompare, ‘CType’ = “tukey-kramer”).

Now, in order to explore global reflectance variation over time, a repeated-measures MANOVA (RM-MANOVA) using the full reflectance spectra (31 wavelengths from 400 to 700 nm at 10 nm intervals) was conducted first. This analysis included the within-subject factor **Time** ($T_{0}$ to $T_{4}$) and the between-subject factors **Material** (polyethylene or polypropylene) and **Condition** (C1 to C4). The response matrix was formed by concatenating all wavelength values for each sample across the five time points.

A complementary multivariate ANCOVA (MANCOVA) was performed to assess the statistical effects of **Material**, **Condition**, **Time** (T1–T4), and their interactions on the average reflectance within each of the seven visible VIS-bands (violet to red). The initial reflectance at $T_{0}$ was included as a covariate to control for baseline differences across samples. Covariates were adjusted via linear regression, and residuals were analyzed through MANOVA. The effects tested included all second-order interactions and the third-order interaction **Material** × **Condition** × **Time**. Multivariate significance was evaluated using Wilks’s λ, Pillai’s trace, Hotelling–Lawley trace, and Roy’s largest root statistics.

Additionally, separate ANCOVA models were fitted for polyethylene and polypropylene to detect potential material-specific effects. Each model included **Condition**, **Time**, the initial reflectance covariate, and their second- and third-order interactions. Univariate *p*-values were used to determine the significance of each term in each VIS-band.

All significance thresholds were set at α=0.05. Results are reported in terms of statistical significance per factor and interaction, and tables summarize the outcomes of all multivariate and adjusted univariate models.

Summarizing this section’s rationale, in order to comprehensively assess the effects of material, exposure condition, and time on spectral reflectance, both univariate and multivariate statistical approaches were employed in this study. Univariate models (ANOVA, ANCOVA) were used to evaluate reflectance changes at each individual VIS-band (e.g., Violet, Blue), allowing for band-specific significance testing. However, given that reflectance values across adjacent VIS-bands are spectrally correlated, multivariate methods (MANOVA, MANCOVA) were also applied to evaluate global degradation patterns. These methods leverage the covariance structure among multiple dependent variables to enhance statistical power, reduce the risk of Type I error associated with multiple testing, and detect interaction effects (e.g., Material × Condition × Time) that may not be evident in isolated univariate analyses. This dual approach enables more robust and interpretable modeling of spectral degradation dynamics across the visible range, as supported by [55], when discussing how multivariate methods can detect global effects that univariate ones may overlook, and [56], by explaining how differences in covariance matrices influence validity and descriptive power features of MANOVAs over ANOVAs when variable dependency could be considered.

### 2.5. Data Processing

Prior to statistical modeling, the raw reflectance measurements were pre-processed to account for replicated wavelengths and categorical structure. Each measurement of reflectance was originally recorded at 21 discrete wavelengths (3 per spectral color) for each sample at each time point.

#### 2.5.1. Reflectance Data

Reflectance was originally recorded at 21 discrete wavelengths for each sample at each time point. These were grouped into seven VIS-bands (Violet to Red), each composed of three closely spaced wavelengths considered as technical replicates. The following steps were taken:

- **Averaging of Technical Replicates.** To minimize redundancy and stabilize the variance, the three reflectance measurements corresponding to each VIS-band (e.g., 400, 410, and 420 [nm] for Violet) were averaged. This yielded one representative reflectance value per color per time point per sample, resulting in a data structure of 7 colors × 5 time points × 32 samples (8 subjects × 4 conditions). These wavelengths within each VIS-band were selected based on spectral proximity (10 nm spacing), forming narrow spectral windows where degradation dynamics are expected to be smooth and correlated. Studies on polymer photo-oxidation and reflectance spectroscopy have consistently shown that UV-induced degradation manifests as gradual, continuous shifts in spectral properties over short wavelength ranges, rather than abrupt changes at narrow intervals [21,41]. Thus, averaging across each band preserves the underlying spectral behavior while improving signal stability. Now, from a statistical perspective, treating each triplet of closely spaced wavelengths as technical replicates reduces random noise and intra-band variability. This not only stabilizes the error variance across repeated-measures and ANCOVA models, but also reinforces key assumptions such as homoscedasticity and normality. Moreover, averaging helps avoid overparameterization in multivariate models, enabling more interpretable and robust estimation of effects across VIS-bands. Consequently, this approach enhances model reliability without compromising spectral resolution in the context of polymer degradation tracking.
- **Reshaping to Longitudinal Format:** The averaged reflectance values were organized into a subject-wise longitudinal data structure across five time points (T0–T4).
- **Covariate Definition:** Reflectance values at T0 were stored separately and used as covariates in ANCOVA and MANCOVA models to adjust for baseline differences in initial color or pigmentation.

#### 2.5.2. Hardness Data

Shore D hardness was measured once at the beginning (T0) and once at the end of the exposure period (T4) for each sample. The data were pre-processed as follows:

- **Delta Hardness Calculation:** For each subject, the change in hardness (ΔHardness = T4 − T0) was computed to quantify mechanical degradation over time.
- **Structuring by Experimental Condition:** The ΔHardness values were grouped by material type and exposure condition to facilitate one-way and two-way ANOVA analyses.

#### 2.5.3. Categorical Encoding

Categorical variables were coded as follows:

- Material: Binary variable (0 = polyethylene, 1 = polypropylene), inferred directly from sample ID.
- Condition: Four-level categorical factor corresponding to exposure condition (C1–C4).
- Time: Five-level within-subject factor (T0–T4), treated as repeated measure.
- Color: Seven-level categorical factor representing the visible spectrum bands (Violet to Red).
- Subject_ID: Unique identifier for each of the 8 distinct subjects (Subject 1 to 8), reused across all conditions.

#### 2.5.4. Dependency Note

It is critical to note that the variables Material and Subject_ID are not statistically independent, as each subject is uniquely associated with either polyethylene or polypropylene. Therefore, in multivariate models involving both factors, collinearity was addressed by excluding one when necessary or by nesting appropriately in hierarchical models.

#### 2.5.5. Software

All preprocessing and statistical modeling were conducted in MATLAB R2024b. Categorical variables were encoded using the categorical() function and all analyses used either built-in functions such as fitrm, manova, fitlm, or custom scripts to handle repeated-measures structures.

## 3. Results and Discussion

### 3.1. Overview of the Dataset

Reflectance data were collected from 32 automotive plastic samples (8 unique subjects × 4 exposure conditions), with each sample measured at 5 time points (T0–T4) and across 31 wavelengths in the visible spectrum (400–700 [nm] at 10 [nm] intervals). Each subject corresponds uniquely to either polyethylene (PE) or polypropylene (PP) and to one specific exposure condition. Reflectance values were pre-processed by averaging the 3 technical replicates per VIS-band when required. The resulting dataset comprises one reflectance value per wavelength, per time point, per sample.

### 3.2. Preliminary Visualization of Reflectance Trends

**Figure 1:**
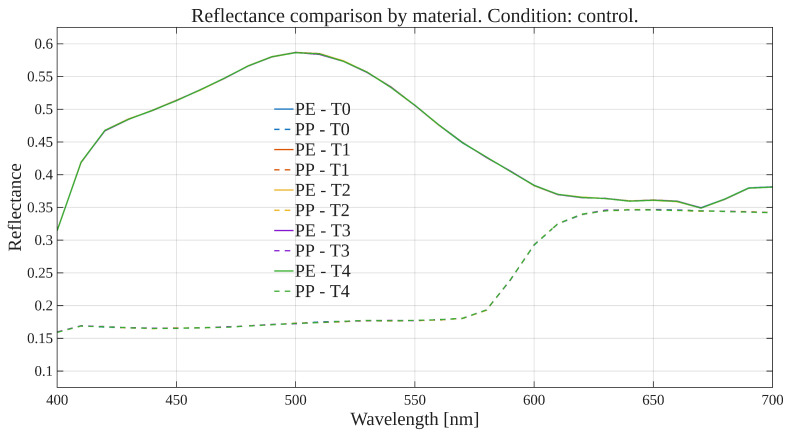
Mean reflectance curves for both polyethylene (solid lines) and polypropylene (dashed lines) under the control condition. One curve per time point (T0–T4).

**Figure 2:**
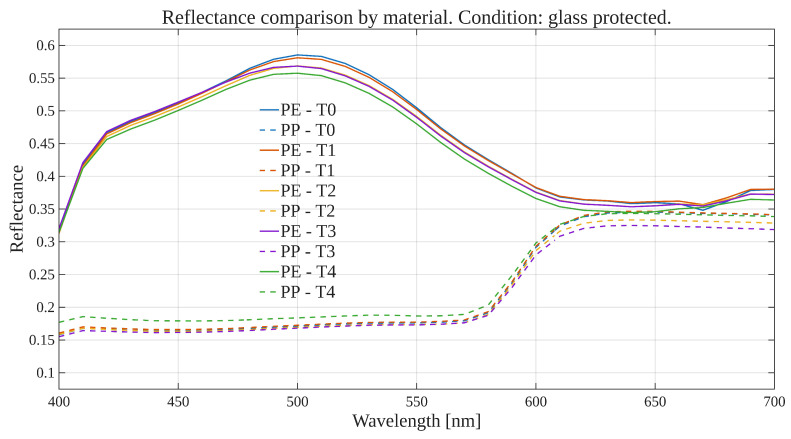
Mean reflectance curves for both polyethylene (solid lines) and polypropylene (dashed lines) under the glass-protected condition. One curve per time point (T0–T4).

**Figure 3:**
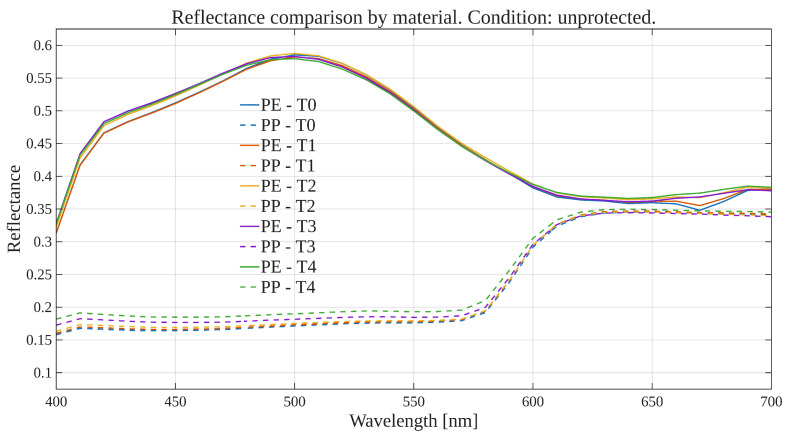
Mean reflectance curves for both polyethylene (solid lines) and polypropylene (dashed lines) under the unprotected condition (no glass coverage). One curve per time point (T0–T4).

**Figure 4:**
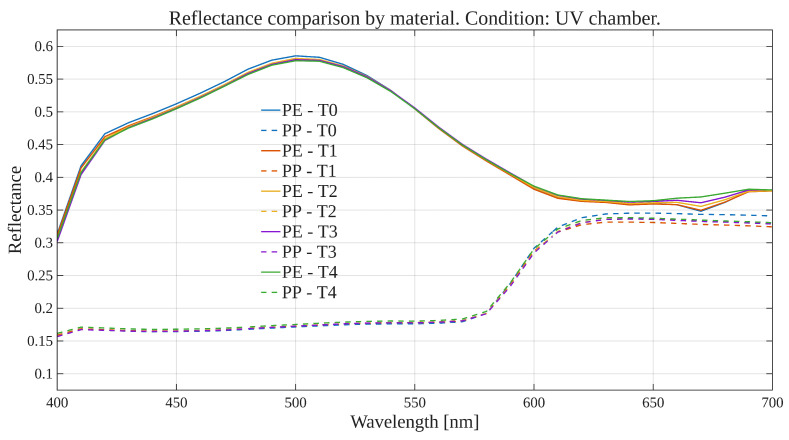
Mean reflectance curves for both polyethylene (solid lines) and polypropylene (dashed lines) under the UV chamber condition (ASTM G154 protocol). One curve per time point (T0–T4).

To explore global reflectance behavior over time and conditions, average reflectance curves were computed for each exposure condition, grouping samples by material (polyethylene or polypropylene). The average reflectance at each wavelength was calculated across all corresponding samples at each time point. Accordingly, Figure 1, Figure 2, Figure 3 and Figure 4 show the average reflectance spectra for both polyethylene (solid lines) and polypropylene (dashed lines) measured at five different exposure times (T0 to T4) under four distinct environmental conditions: no exposure, glass-covered exposure, uncovered exposure, and accelerated exposure in a UV chamber (ASTM G154 protocol). Consequently, each figure presents the evolution of reflectance as a function of wavelength (400–700 [nm]), grouped by material and time.

Under control conditions (Figure 1), both PE and PP maintain stable reflectance profiles across the spectrum throughout the entire exposure period. No significant shifts or trends are observed, confirming this condition as a reliable baseline. Under the glass-protected exposure condition (Figure 2), PE exhibits decrement reflectance variation over time, more distinguishable from 470 [nm] on to longer wavelengths. In the same condition, PP shows distinguishable variations but without a well-defined trend over time. These patterns suggest the presence of photo-oxidative effects, possibly mitigated by the glass barrier, that are more prevalent for PE than PP. On the other hand, in the unprotected condition (Figure 3), PE showed reflectance variation with no definite tendency over time from the entire VIS-band. In contrast, PP data allows for the observation of an incremental variation in reflectance from T0 to T4, prevalent at shorter wavelengths (specially from 400 to 580 [nm] approximately). Finally, regarding the UV chamber condition (Figure 4), PE reflectance variation followed a decremental trend for shorter wavelengths (400 to 520 [nm] approximately), followed by an incremental one at longer wavelengths (660 to 700 [nm]). Meanwhile, PP shows reflectance variations with no discernible trend along the entire VIS-band.

Overall, polyethylene exhibited higher reflectance values across all wavelengths and conditions compared to polypropylene. In both materials, the most noticeable changes occurred under the glass-protected and unprotected exposure conditions. These findings indicate that material type and exposure condition influence the optical behavior of the polymers over time.

On the other hand, these visualizations serve as an initial qualitative inspection of reflectance variation/degradation over time, revealing both wavelength-dependent and material-dependent differences, and motivating the subsequent statistical analysis aimed at formally quantifying these effects through univariate and multivariate statistical models present in the following sections.

### 3.3. Temporal Evolution by VIS-Band and Exposure Condition

In order to examine how surface optical properties evolve under different exposure conditions, reflectance trends were analyzed across seven visible VIS-bands. The analysis compares polyethylene and polypropylene behaviors over five time points (T0–T4) under four exposure conditions.

**Figure 5:**
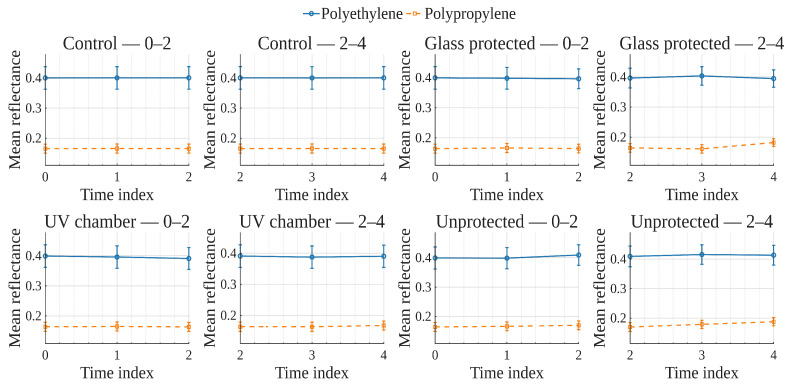
Temporal evolution of mean reflectance in the Violet band (400–420 nm) for polyethylene (solid lines) and polypropylene (dashed lines) under four conditions (Control, Glass-protected, Unprotected, and UV chamber). Panels split the time course into early (0–2) and late (2–4) intervals; markers denote integer times (T0–T4). Error bars show visually scaled (×0.30) SEM—standard error of the mean—applied uniformly across panels.

**Figure 6:**
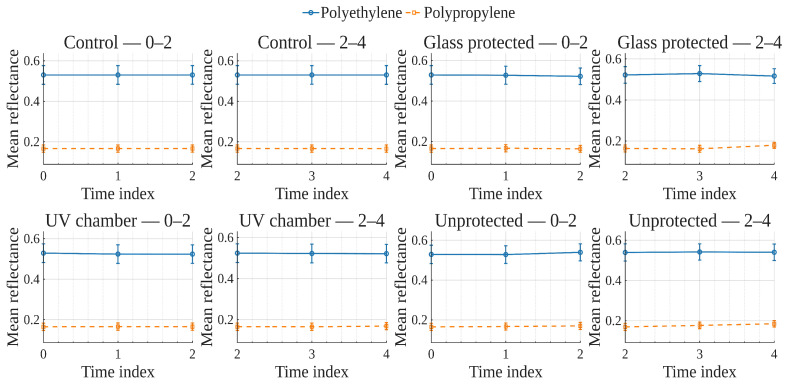
Temporal evolution of mean reflectance in the Blue band (450–470 nm) for polyethylene (solid) and polypropylene (dashed) across the four conditions (Control, Glass-protected, Unprotected, and UV chamber). Panels split the time course into early (0–2) and late (2–4) intervals; markers denote integer times (T0–T4). Error bars show visually scaled (×0.30) SEM—standard error of the mean—applied uniformly across panels.

**Figure 7:**
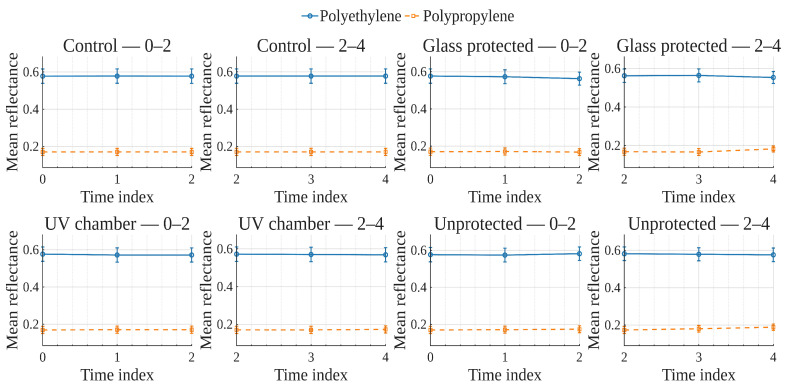
Temporal evolution of mean reflectance in the Cyan band (480–500 nm) comparing polyethylene (solid) versus polypropylene (dashed) under Control, Glass-protected, Unprotected, and UV chamber conditions. Panels split the time course into early (0–2) and late (2–4) intervals; markers denote integer times (T0–T4). Error bars show visually scaled (×0.30) SEM—standard error of the mean—applied uniformly across panels.

- Violet (400–420 [nm]), Blue (450–470 [nm]), and Cyan (480–500 [nm]) bands (Figure 5, Figure 6, Figure 7).

In the **UV chamber** condition, both PE and PP exhibited minimal variation in reflectance across time. Under **unprotected** exposure, PE showed a noticeable increase, particularly between T3 and T4, whereas PP remained stable. For the **control** condition, reflectance remained steady for both materials throughout the test period, whereas for the **glass-protected** condition, slight decreases and increases for PE and PP are observed, respectively.

**Figure 8:**
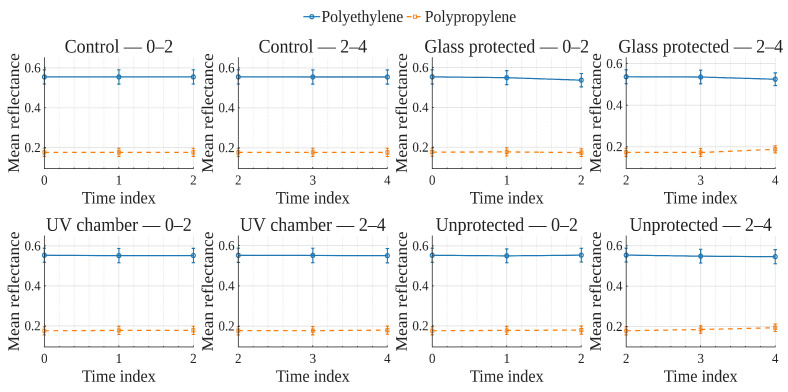
Temporal evolution of mean reflectance in the Green band (520–540 nm) for polyethylene (solid) and polypropylene (dashed) under the four illumination/exposure conditions. Panels split the time course into early (0–2) and late (2–4) intervals; markers denote integer times (T0–T4). Error bars show visually scaled (×0.30) SEM—standard error of the mean—applied uniformly across panels.

**Figure 9:**
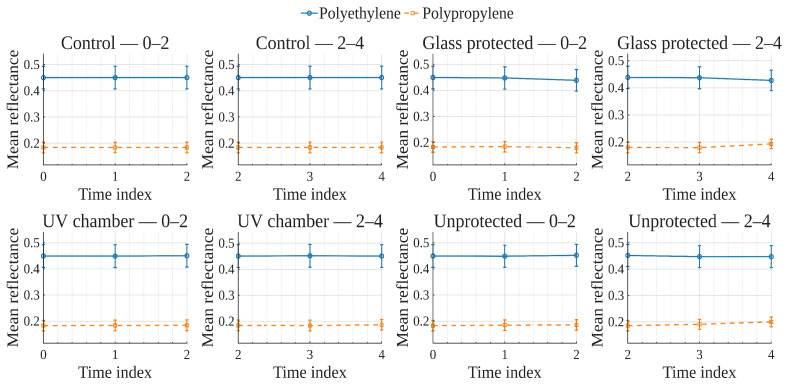
Temporal evolution of mean reflectance in the Yellow band (560–580 nm) for polyethylene (solid) and polypropylene (dashed), by condition (Control, Glass-protected, Unprotected, and UV chamber). Panels split the time course into early (0–2) and late (2–4) intervals; markers denote integer times (T0–T4). Error bars show visually scaled (×0.30) SEM—standard error of the mean—applied uniformly across panels.

- Green (520–540 [nm]), and Yellow (560–580 [nm]) bands (Figure 8 and Figure 9).

Under the **UV chamber** condition, PE shows minimal (almost indistinguishable in the image) decrease, while that was not the case for PP, with no apparent variation. Regarding **unprotected** exposure, PP does not show noticeable change, but PE showed an increase between T3 and T4. For the **control** condition, reflectance did not noticeably change for either material throughout the test period. Simultaneously, for the **glass-protected** condition, slight variations for PE and PP are observed, but with no overall discernible reflectance increase or decrease trend.

**Figure 10:**
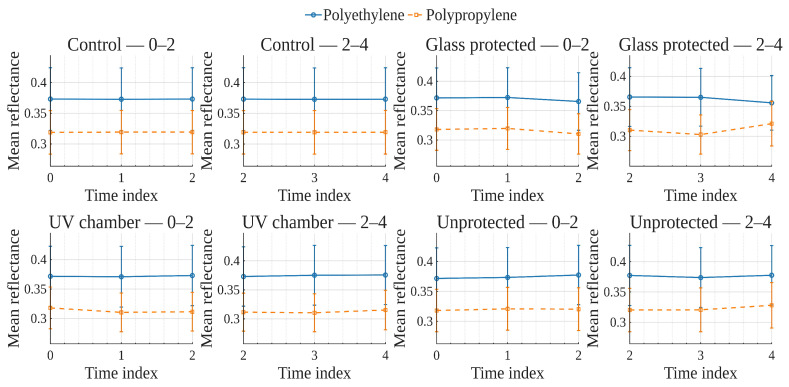
Temporal evolution of mean reflectance in the Orange band (600–620 nm) for polyethylene (solid) versus polypropylene (dashed), shown per condition. Time segmentation (0–2 vs. 2–4) and scaled (×0.30) SEM—standard error of the mean bars.

**Figure 11:**
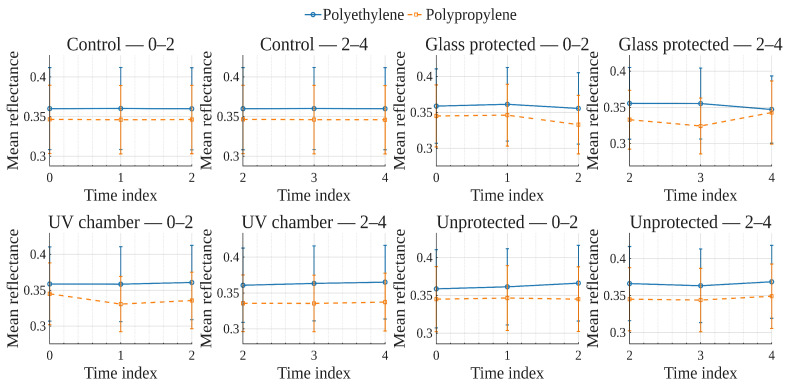
Temporal evolution of mean reflectance in the Red band (640–660 nm) contrasting polyethylene (solid) and polypropylene (dashed) under Control, Glass-protected, Unprotected, and UV chamber conditions. Panels: 0–2 and 2–4; intervals: markers at integer times. Error bars: scaled SEM (×0.30) applied uniformly across bands and conditions.

- Orange (600–620 [nm]) and Red (640–660 [nm]) bands (Figure 10 and Figure 11).

**UV chamber** condition shows no variation for either material. **Unprotected** exposure depicts variation for PE, but no definite trend towards increase or decrease, and no change for PP. Now, for the **control** condition, reflectance did not show noticeable change for either material throughout the test period. With regards to the **glass-protected** condition, no variation is discernible for PP. In contrast, very small variations are detectable for PE, but with no definite trend for the Orange band and a very small incremental trend for the Red band.

In summary, due to the relative scale used in these previously discussed visualizations (Figure 5, Figure 6, Figure 7, Figure 8, Figure 9, Figure 10 and Figure 11), which is necessary to allow for material comparison/joint-visualization, variations are arduous to observe. However, polyethylene consistently demonstrated more sensitivity to environmental exposure—particularly in glass-protected and unprotected conditions—through gradual reflectance changes, especially in shorter wavelengths. In contrast, polypropylene remained more stable across all bands and conditions, highlighting its higher resistance to photodegradation under the tested scenarios.

### 3.4. Time Evolution of Subject-Normalized Degradation Efficiency

**Figure 12:**
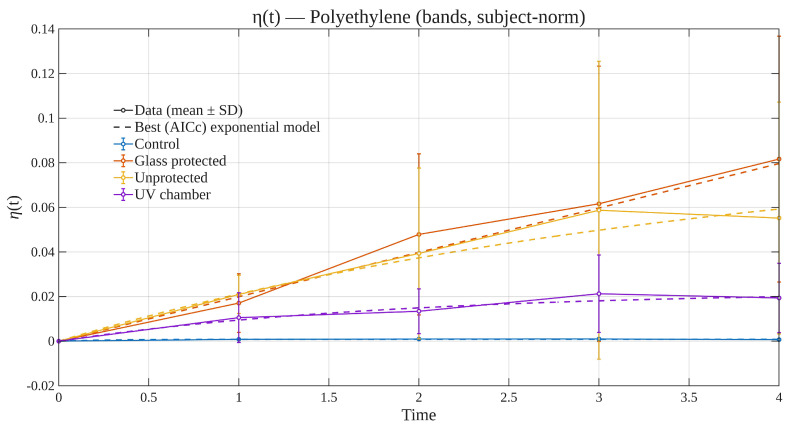
Subject-normalized degradation efficiency η(t) for **polyethylene** computed from spectral reflectance as $\eta_{i}\left(t\right)={∥R_{i}\left(t\right)-R_{i}\left(t_{0}\right)∥}_{2}/{∥R_{i}\left(t_{0}\right)∥}_{2}$, and then averaged across specimens within each condition (markers show group mean; error bars denote ±SD). Dashed curves are the best exponential fits selected by AICc among the candidate family (first-order, stretched, lag/offset variants, and bi-exponential). Time is shown as the discrete sampling index ($t_{0}$–$t_{4}$).

**Figure 13:**
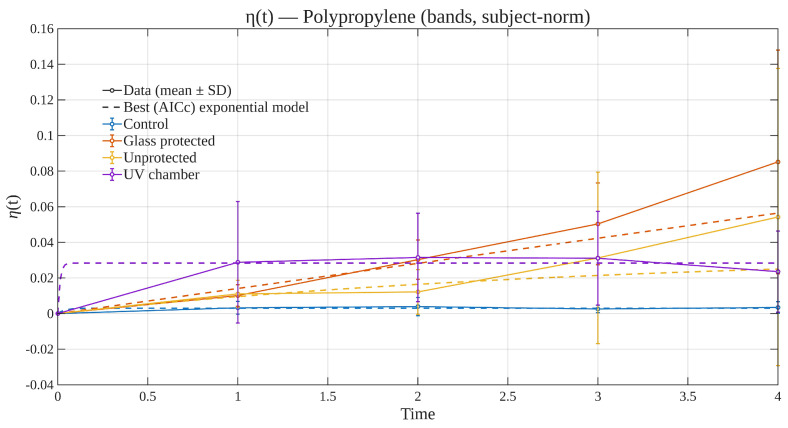
Subject-normalized degradation efficiency η(t) for **polypropylene** under the four exposure conditions (mean ± SD across specimens). Dashed lines indicate the AICc-selected best exponential model in each condition. Time is the discrete sampling index ($t_{0}$–$t_{4}$).

Figure 12 and Figure 13 show the temporal evolution of the subject-normalized degradation efficiency, η(t), computed on seven reflectance bands (three wavelengths per band, as described in Section 2.1) for PE and PP. For each material–condition–time cell, the reflectance of each individual specimen was first normalized to its own baseline spectrum at t=0 (per-specimen $\left|\right|R_{0}\left|\right|$ normalization across the relevant bands), computed the per-specimen efficiency $\eta_{i}\left(t\right)$, and then aggregated across specimens to obtain the mean and uncertainty at each time point. In detail, η(t) is defined in terms of the reflectance vectors measured at multiple wavelengths. For each specimen *i* (piece), material *m*, and exposure condition *c*, $R_{i}\left(t_{k},\lambda_{j}\right)$ is the reflectance at time index $t_{k}$ and wavelength $\lambda_{j}$ (j=1,…,J). The specimen’s own first measurement was taken as its baseline,

$$t_{0,i}=\min\left\{t_{k}\;\mathrm{observed}\;\mathrm{for}\;\mathrm{specimen}\;i\right\},\;R_{0,i}=\left[R_{i}\left(t_{0,i},\lambda_{1}\right),\ldots ,R_{i}\left(t_{0,i},\lambda_{J}\right)\right].$$

Defining the $\ell_{2}$ norm ${∥x∥}_{2}={\left(\sum_{j}x_{j}^{2}\right)}^{1/2}$, the subject-normalized efficiency for specimen *i* is

(1) $$\eta_{i}\left(t_{k}\right)=\frac{{∥R_{i}\left(t_{k}\right)-R_{0,i}∥}_{2}}{{∥R_{0,i}∥}_{2}},\;R_{i}\left(t_{k}\right)=\left[R_{i}\left(t_{k},\lambda_{1}\right),\ldots ,R_{i}\left(t_{k},\lambda_{J}\right)\right].$$

This definition uses all *J* wavelengths (J=31) measured with the spectrophotometer. Now, in order to obtain a color-banded summary, wavelengths were grouped into seven triplets $B_{b}$ (violet, blue, cyan, green, yellow, orange, red) and averaged within each band:

$${\bar{R}}_{i}\left(t_{k},b\right)=\frac{1}{|B_{b}|}\sum_{\lambda \in B_{b}}R_{i}\left(t_{k},\lambda \right),\;{\bar{R}}_{i}\left(t_{k}\right)=\left[{\bar{R}}_{i}\left(t_{k},1\right),\ldots ,{\bar{R}}_{i}\left(t_{k},7\right)\right],$$

$\eta_{i}\left(t_{k}\right)$ was defined as in (1), but replacing $R_{i},R_{0,i}$ by their band-averaged versions ${\bar{R}}_{i},{\bar{R}}_{0,i}$. These last are the ones presented in Figure 12 and Figure 13. For each material–condition group (m,c), specimens were aggregated at each time $t_{k}$:

(2) $${\bar{\eta }}_{m,c}\left(t_{k}\right)=\frac{1}{n_{m,c}\left(t_{k}\right)}\sum_{i\in (m,c)}\eta_{i}\left(t_{k}\right),\;s_{m,c}\left(t_{k}\right)=\sqrt{\frac{1}{n_{m,c}\left(t_{k}\right)-1}\sum_{i\in (m,c)}{\left(\eta_{i}\left(t_{k}\right)-{\bar{\eta }}_{m,c}\left(t_{k}\right)\right)}^{2}}.$$

Values ${\bar{\eta }}_{m,c}\left(t_{k}\right)$ were plotted with error bars chosen as either SD ($\pm s_{m,c}$), SEM ($\pm s_{m,c}/\sqrt{n}$), or a 95% confidence interval ($\pm 1.96\;s_{m,c}/\sqrt{n}$). The error bars presented in Figure 12 and Figure 13 are the first choice.

- *Kinetic models and parameter interpretation.*

${\bar{\eta }}_{m,c}\left(t\right)$ values were fit with physically interpretable monotonic exponentials (nonlinear least squares) using SD-based weights $w_{k}=1/s_{m,c}{\left(t_{k}\right)}^{2}$ when available. Candidate models considered were

(3) $$\begin{array}{cccc} \mathrm{First}\text{-}\mathrm{order}:\; & \eta \left(t\right)=\eta_{\infty }\;\left(1-e^{-t/\tau }\right), & \; & \eta_{\infty }\geq 0,\;\tau >0, \end{array}$$

(4) $$\begin{array}{cccc} \mathrm{Stretched}:\; & \eta \left(t\right)=\eta_{\infty }\;\left(1-e^{-{\left(t/\tau \right)}^{\beta }}\right), & \; & \eta_{\infty }\geq 0,\;\tau >0,\;0.2\leq \beta \leq 2.5, \end{array}$$

(5) $$\begin{array}{cccc} \mathrm{First}\text{-}\mathrm{order}\;\mathrm{with}\;\mathrm{lag}:\; & \eta \left(t\right)=\eta_{\infty }\;\left(1-e^{-\max(t-\delta ,0)/\tau }\right), & \; & \delta \geq 0, \end{array}$$

(6) $$\begin{array}{cc} \mathrm{Stretched}\;\mathrm{with}\;\mathrm{lag}:\; & \eta \left(t\right)=\eta_{\infty }\;\left(1-e^{-{\left(\max(t-\delta ,0)/\tau \right)}^{\beta }}\right), \end{array}$$

(7) $$\begin{array}{cccc} \mathrm{First}\text{-}\mathrm{order}\;\mathrm{with}\;\mathrm{offset}:\; & \eta \left(t\right)=\eta_{0}+\eta_{\infty }\;\left(1-e^{-t/\tau }\right), & \; & \eta_{0}\in \left[-0.05,0.05\right]. \end{array}$$

Half-times can be considered directly. For Equations (3)–(7),

$$t_{1/2}=\tau \ln2\;\;(\mathrm{no}\;\mathrm{lag}),\;\;t_{1/2}=\delta +\tau \ln2\;\;(\mathrm{with}\;\mathrm{lag}),\;\;and\;\;t_{1/2}=\tau {\left(\ln2\right)}^{1/\beta }\;\mathrm{for}\;\mathrm{stretched}\;\mathrm{forms}.$$

- *Model selection (AICc).*

For each candidate with *k* free parameters, the weighted residual sum of squares is

$$\mathrm{SSE}=\sum_{k=1}^{n}w_{k}{\left[{\bar{\eta }}_{m,c}\left(t_{k}\right)-\hat{\eta }\left(t_{k}\right)\right]}^{2},$$

and the following computation was performed:

$$\mathrm{AIC}=n\ln\;\left(\frac{\mathrm{SSE}}{n}\right)+2k,\;{\mathrm{AIC}}_{c}=\mathrm{AIC}+\frac{2k(k+1)}{n-k-1}.$$

The model with the smallest ${\mathrm{AIC}}_{c}$ was reported as “best” and drawn as the main dashed curve in the overlays shown by Figure 12 and Figure 13.

- *Interpretation.*

Since η(t) measures the *relative* spectral distance from each piece’s own baseline (Equation (1)), values near zero indicate minimal change; larger values quantify stronger spectral evolution (e.g., due to oxidation, yellowing, or microcracking). Subject normalization removes differences (such as color and finish, which were not an issue from the get-go) at t=0 and makes groups comparable; error bars reflect between-piece dispersion within each material–condition–time cell and directly inform weighting in the fits. Within Figure 12 and Figure 13, dashed curves correspond to the best exponential model selected by AICc among a small family with physical interpretability (first-order, stretched exponential, and lag/offset variants), fitted to the band-wise mean trajectories with optional inverse-variance weighting; solid symbol–lines connect the observed means for readability. For both materials, the *Control* condition remains essentially flat at η(t)≈0 over the 4-month window, as expected. The *UV chamber* condition exhibits a modest early increase followed by a shallow plateau, consistent with a rapid initial response and limited cumulative dose. In contrast, the outdoor *Unprotected* and *Glass-protected* conditions show monotonic growth of η(t), with *Glass-protected* typically presenting the largest values at each time point. This ordering is consistent with a scenario where the glass filter modifies the incident spectrum and thermal load, yielding measurable—but still bounded—cumulative change over the study period. Across both materials, the AICc favored the simple first-order model in all band-wise cases presented, indicating that over this limited exposure interval the data are well described by a single characteristic timescale without requiring additional stretching or lag terms. Taken together, these trajectories indicate negligible drift in controls, modest chamber-induced change, and progressive outdoor degradation with spectral filtering having a measurable effect.

- *Related context to prior works.*

Table 3 situates the present study within representative literature on polyolefin degradation. Prior studies typically quantify ageing using chemical (e.g., FTIR carbonyl index—CI), optical (UV-VIS color/yellowness), or mechanical proxies and then discuss trend shapes qualitatively or with single-model fits tied to specific test setups. In contrast, this study systematically links broadband optical response to time-resolved damage across multiple illuminations and for two different materials, and does so with a unified, bounded response variable and a transparent model-selection protocol. Concretely, a time series of broadband reflectance (both full-spectrum and seven VIS-bands) is analyzed and fitted into physically interpretable kinetics (first-order, stretched, lagged, and bi-exponential), selecting the description by AICc with uncertainty-weighted regression. This yields directly comparable summary parameters (e.g., $t_{1/2}$, asymptotic response) across conditions, enabling the possibility of cross-material and cross-illumination ranking rather than case-by-case narratives. In particular, regarding the subject of degradation efficiency, while previous reports often emphasize a single proxy and a single kinetic hypothesis, the present framework (i) treats the optical signal as an *integrated* damage reporter over the spectrum (not merely a narrow spectral index), (ii) compares several mechanistic exponentials under the same informational/theoretical framework, and (iii) provides compact visual summaries (such as overlays and heatmaps (these latter not included currently in the manuscript) that indicates when simple first-order behavior suffices and when multi-timescale (bi-exponential) dynamics could be more appropriate. For completeness, the bounded response was denoted as η(t)∈[0,1] to summarize progression and was conceived primarily as a convenient reporting scale rather than as a claim of main contribution.

In summary, the approach presented in this work with respect to prior polyolefin studies—predominantly relying on chemical/color indices (e.g., carbonyl index, yellowness), CO_2_ evolution, or mechanical retention—contrasts with the tracking of time-resolved visible reflectance aggregated into seven VIS-bands and summarizing of its evolution through a bounded, model-based trajectory η(t). This representation provides a compact, visually comparable signature across materials and exposures without requiring destructive assays. We restrict η(t) to graphical summaries—consistent with the scope of the study—and defer tabulated kinetic constants (e.g., $t_{1/2},\eta_{\infty }$) to future, higher-powered datasets. This keeps the emphasis on comparability (PE vs. PP; outdoor vs. glass-protected vs. UV chamber) while maintaining methodological consistency.

### 3.5. Effect of Environmental Exposure on Shore D Hardness

The statistical analysis of Shore D hardness summarized in Table 4 portrays significant degradation in both materials under specific exposure conditions. For polyethylene, paired hypothesis tests showed significant decreases in hardness after exposure to outdoor conditions without glass protection (OEW/OG) and to the UV chamber (UVC), while no significant change was observed under outdoor exposure with glass (OEWG). In polypropylene, all post-exposure conditions led to statistically significant hardness losses relative to the reference state (RH), indicating higher susceptibility to both direct and filtered UV exposure.

**Figure 14:**
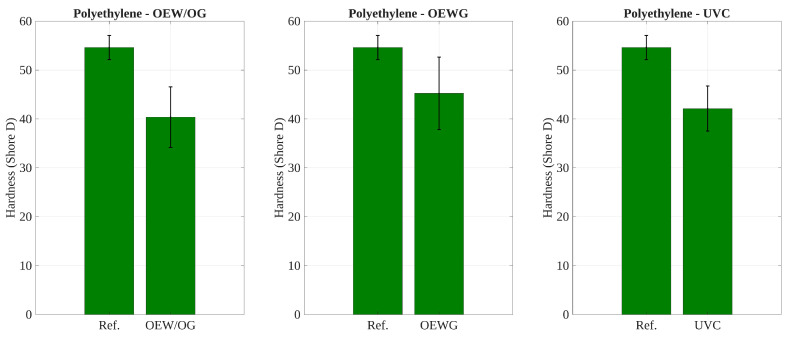
Hardness comparison for polyethylene components before and after exposure. Bar plots show mean Shore D hardness with standard deviation. Three exposure conditions are compared against the reference: OEW/OG (open environment without glass), OEWG (open environment with glass), and UVC (simulated ultraviolet exposure). Significant differences are highlighted from pairwise tests.

**Figure 15:**
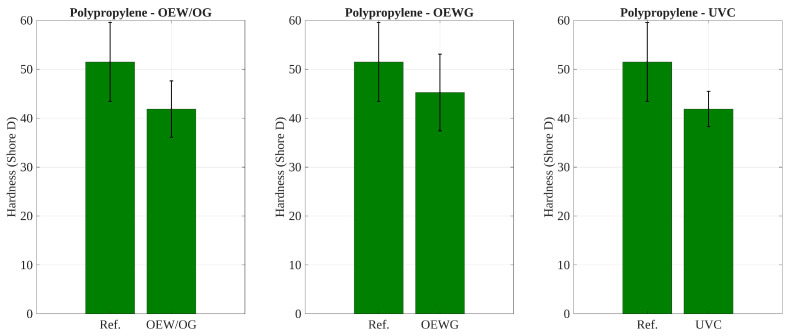
Hardness comparison for polyethylene components before and after exposure. Bar plots show mean Shore D hardness with standard deviation. Three exposure conditions are compared against the reference: OEW/OG (open environment without glass), OEWG (open environment with glass), and UVC (simulated ultraviolet exposure). Significant differences are highlighted from pairwise tests.

On the other hand, the one-way ANOVA confirmed overall differences among conditions for polyethylene (p<0.05), with Tukey’s test identifying significant pairwise differences between the reference and the OEW/OG and UVC groups. In contrast, for polypropylene, the ANOVA result was not statistically significant (p>0.05), likely due to larger within-group variability; however, the post hoc test still detected consistent pairwise differences between the reference and all exposure conditions. In detail, as shown in Table 4, while all pairwise comparisons for polypropylene samples showed significant differences (p<0.05), the global one-way ANOVA test did not reach statistical significance (p>0.05). This arises due to the inherent differences between these two statistical approaches. Pairwise tests compare each post-exposure condition to the reference individually, using fewer degrees of freedom while being sensitive to small effect sizes. In contrast, the ANOVA global test evaluates the variance among all groups simultaneously, where high within-group variability and relatively small sample sizes can reduce statistical power. Additionally, since ANOVA relies on the overall variance structure, it may fail to detect significance when the differences are not uniformly distributed across all groups. For clarity, standard deviation error bars have been added to Figure 14 and Figure 15 to visually illustrate the within-group variability in Shore D hardness for polypropylene. This visualization supports the statistical findings and helps explain why the ANOVA test did not reach significance despite consistent pairwise results.

These results indicate that polypropylene is more vulnerable to hardness loss under environmental exposure, including when protected by glass, whereas polyethylene exhibits partial resilience when shielded from direct UV radiation.

### 3.6. Material Non-Specific ANOVA and ANCOVA

#### 3.6.1. Effect of Material, Condition, and Time on Reflectance: Three-Way ANOVA

The ANOVA results show that the factor **Material** had a statistically significant effect (p<0.05) on average reflectance across the spectrum, particularly for the shorter wavelength bands (Violet to Yellow). This indicates that polyethylene and polypropylene differ systematically in their optical response under environmental exposure.

No statistically significant effects (p>0.05) were found for the factors **Condition** or **Time**, nor for any second- or third-order interaction terms. These results suggest that, when pooling all materials together, neither the specific exposure condition nor the measurement time produced detectable differences in reflectance within each VIS-band.

It is noteworthy that only the material type consistently influenced reflectance for the first five VIS-bands (Violet to Yellow), while no significant effects were detected in the longer wavelength regions (Orange and Red). These findings are consistent with the physical expectation that UV-induced degradation may manifest more strongly in the short-wavelength reflectance behavior.

#### 3.6.2. Controlling for Baseline Differences: Full-Factorial ANCOVA Model

Table 5 summarizes the ANCOVA significance outcomes for each visible VIS-band, considering the effects of **Material**, **Condition**, **Time**, and the covariate *initial reflectance*, as well as their interactions up to the four-way level.

The covariate was statistically significant (p<0.05) in all seven VIS-bands, confirming that baseline differences in reflectance strongly influence the response during exposure. Condition exhibited a significant effect in six out of seven bands (all except Orange), indicating that environmental exposure type plays a central role in the reflectance degradation process. Time was also significant in Violet, Blue, Cyan, and Yellow, suggesting that degradation dynamics evolve significantly over time, especially in the shorter wavelengths of the visible spectrum.

The effect of Material was only significant in the Blue and Cyan bands, implying that polyethylene and polypropylene exhibit distinct degradation behavior primarily in the central spectral range. Among the interaction terms, Condition × Covariate was significant in all bands, pointing to a dependency of reflectance response on both baseline reflectance and environmental scenario. Similarly, Time × Covariate interactions were significant in Blue, Cyan, Green, and Yellow, highlighting that the rate or direction of change over time is modulated by initial reflectance values.

The Material × Condition interaction reached significance in Blue, Orange, and Red, suggesting that the effect of exposure condition differs between materials, particularly at the spectral extremes. Moreover, third-order interactions involving the covariate, specifically Material × Condition × Covariate and Material × Time × Covariate, were significant in the Orange and Red bands, pointing towards potential complex dependencies at longer wavelengths. However, no four-way interaction (Material × Condition × Time × Covariate) was significant in any VIS-band, indicating that the combined effect of all experimental factors and the covariate does not significantly explain additional variance beyond the lower-order terms.

Overall, Blue and Cyan emerged as the most responsive VIS-bands to both main effects and interactions, while Orange and Red exhibited more nuanced, higher-order interaction patterns. These findings underscore the importance of considering spectral specificity and covariate adjustment when modeling reflectance degradation in polymeric materials exposed to environmental stressors.

### 3.7. Material Specific ANOVA and ANCOVA

#### 3.7.1. Separate Effects of Exposure and Time Within Each Material: Two-Way ANOVA

The two-way ANOVA results summarized in Table 6 indicate that none of the evaluated factors—Condition, Time, or their interaction—had a statistically significant effect on the average reflectance within any visible VIS-band (*p* > 0.05 in all cases). This finding held consistently across both material types, polyethylene (PE) and polypropylene (PP). These results suggest that, for the considered statistical framework, under the current experimental conditions and timescale, the reflectance of the tested automotive plastic specimens remained stable regardless of the type of environmental exposure or the duration of the exposure period.

In order to explore that perspective, three steps were taken for each band and material in Table 6 with the interaction model *Condition*, *Time*, *Condition* × *Time*. First, eta-squared (*partial effect size*) and Cohen’s $f^{2}$, were calculated, respectively:

(8) $$\eta_{p}^{2}\;=\;\frac{{\mathrm{SS}}_{\mathrm{effect}}}{{\mathrm{SS}}_{\mathrm{effect}}+{\mathrm{SS}}_{\mathrm{error}}}$$

(9) $$f^{2}\;=\;\frac{\eta_{p}^{2}}{1-\eta_{p}^{2}}$$

Second, to relate non-significant results to sensitivity, a post hoc power analysis for a small “smallest effect size of interest” (SESOI) $f^{2}=0.02$ (Cohen’s convention), with α=0.05 and target power 1−β=0.80 was also performed. Power for each test was calculated under the noncentral *F* distribution with noncentrality

(10) $$\lambda \;=\;f^{2}\;\cdot \;df_{\mathrm{error}},$$

so that

(11) $$\mathrm{Power}\;=\;1-F_{\mathrm{nc}F}\;\left(F_{1-\alpha };\;df_{1},\;df_{2},\;\lambda \right),$$

where $F_{\mathrm{nc}F}\left(\;\cdot \;;\;df_{1},df_{2},\lambda \right)$ is the (noncentral) cumulative distribution function (CDF) of *F*, $F_{1-\alpha }$ is the (1−α) critical value of the central $F(df_{1},df_{2})$ with numerator and denominator degrees of freedom $df_{1}$ and $df_{2}$ respectively, and λ is the noncentrality parameter. Here, $df_{1}$ corresponds to the effect’s degrees of freedom (e.g., levels of *Condition*, *Time*, or their interaction minus one), and $df_{2}$ corresponds to the model’s error degrees of freedom. The noncentrality parameter λ quantifies the true signal strength relative to noise. As previously mentioned, under the general linear model (GLM) formulation used, $\lambda =f^{2}\;\cdot \;df_{\mathrm{error}}$.

Third, the minimum detectable effect (MDE) $f^{2}$ at 80% power was also reported by numerically solving the above expression for $f^{2}$:

(12) $${\mathrm{MDE}}_{0.80}\;=\;\min\left\{f^{2}:\;\mathrm{Power}\left(f^{2}\right)\geq 0.80\right\}.$$

Time was treated as categorical (equally spaced levels but not assumed linear), matching Table 6. In this context, $\eta_{p}^{2}$ gauges the share of variance uniquely attributable to a term after partialling out the error term; $f^{2}$ rescales $\eta_{p}^{2}$ to a signal-to-noise metric useful for power.

Results indicated that across bands, eta-squared (*partial effect size*) estimates for *Condition*, *Time*, and their interaction were near zero (typical medians ≤0.001). For the SESOI $f^{2}=0.02$, post-hoc power was below 0.80 for all terms. Consequently, the median *MDE* at 80% power was ≈0.19 for *Condition*, ≈0.22 for *Time*, and ≈0.34 for the interaction (values similar in PE and PP). In summary, these results indicate that the analysis behind Table 6 (two-way ANOVA with Time categorical and omnibus tests) is less sensitive to small effects, since the design would have reliably detected medium–large effects (MDEs ≈0.19–0.34), but not very small ones. As the observed $\eta_{p}^{2}$ are uniformly tiny, the null results are consistent with the absence of medium/large effects rather than with broad under-sampling. On the other hand, this interpretation also aligns with other sections of the present manuscript, since alternative analyses did identify effects. Thus, it is within reason considering that the dataset is not generally under-powered, but that the specific Table 6 framework is inherently insensitive to very small effects.

#### 3.7.2. Material-Specific ANCOVA: Separate Models for Polyethylene and Polypropylene

Table 7 presents the ANCOVA results for each VIS-band analyzed separately by material. For **polyethylene**, the covariate (initial reflectance) was consistently significant (p<0.05) across all VIS-bands, indicating strong baseline dependence in the observed reflectance patterns. Condition was also significant in all bands, whereas Time showed significance only in the blue band. Notably, interactions involving the covariate were frequent: Condition × Covariate and Time × Covariate were both significant in seven, six out of seven bands respectively, and the three-way interaction (Condition × Time × Covariate) appeared only in yellow and orange.

In contrast, for **polypropylene**, only the covariate was significant in the first five VIS-bands (Violet to Yellow), with no significant effects of Condition or Time, except for Orange and Red in the former, nor any interactions in those bands. Significant effects of Condition and Condition × Covariate emerged only in the Orange and Red bands, accompanied by a significant Time × Covariate interaction in Orange. This suggests that reflectance variation in polypropylene is less sensitive to experimental factors than in polyethylene and largely governed by initial reflectance levels except at longer wavelengths.

Overall, the results indicate material-dependent reflectance behavior: Polyethylene shows broader sensitivity to environmental exposure and time effects, while polypropylene’s variation is more restricted and primarily driven by baseline properties.

### 3.8. Material Non-Specific MANOVA and MANCOVA

#### 3.8.1. Multivariate Assessment of Experimental Factors on Reflectance Response: Full MANOVA Model

As shown in Table 8, a multivariate analysis of variance (MANOVA) was conducted in order to evaluate the effects of *Material*, *Condition*, *Time*, and their interactions on the multivariate response of average reflectance across seven VIS-bands in the visible spectrum (Violet, Blue, Cyan, Green, Yellow, Orange, Red). The analysis showed that only the main effect of *Material* was statistically significant (p<0.05) across all multivariate test statistics (Wilks’s λ, Pillai’s trace, Hotelling–Lawley trace, and Roy’s greatest root). In contrast, neither *Condition*, nor *Time*, nor any of their two-way or three-way interactions with *Material*, showed significant effects (p>0.05). These results indicate that the material type (polyethylene or polypropylene) significantly influences the overall reflectance profile, independently of the environmental exposure conditions or duration of exposure.

#### 3.8.2. MANCOVA Analysis of Visible Reflectance Adjusted by Initial Conditions

The MANCOVA results adjusted by initial reflectance (T0) in Table 9 reveal that none of the main effects—**Material**, **Condition**, or **Time**—were statistically significant according to any of the four multivariate test statistics. Among the second-order interactions, only **Material × Condition** showed significance under the Hotelling–Lawley trace, suggesting a possible dependence of reflectance variation on the specific combination of material and environmental exposure. The third-order interaction **Material × Condition × Time** was also statistically significant under the Hotelling–Lawley criterion, indicating that temporal changes in reflectance may be differentially affected depending on both material type and exposure condition. These significances must be considered within the context of other multivariate statistics such as the ones also discussed in this manuscript, needing thoughtful interpretation.

### 3.9. Material-Specific MANOVA and MANCOVA

#### 3.9.1. Repeated-Measures MANOVA: Within-Material Analysis of Exposure and Time

The multivariate results in Table 10 show that, for polyethylene, the factor *Condition* had a statistically significant effect on the mean reflectance across the visible spectrum, as evidenced by all four multivariate tests (p<0.05). In contrast, for polypropylene, no consistent multivariate significance was found for *Condition*, although Roy’s test did indicate marginal significance. For both materials, the factor *Time* and the interaction *Condition × Time* were not significant according to Wilks’s, Pillai’s, and Hotelling–Lawley criteria; however, Roy’s test did detect significance for *Time* in both materials and for the interaction in polyethylene. This pattern suggests that differences between exposure conditions had a more pronounced and consistent effect on polyethylene reflectance, whereas time-dependent changes were weaker and dependent on the test statistic used.

#### 3.9.2. Separate MANCOVA Models by Material Adjusted for Initial Reflectance

The MANCOVA results in Table 11 adjusted by initial reflectance (T0) and stratified by material reveal distinct multivariate response patterns between polyethylene and polypropylene samples. For **polyethylene**, the effects of *Condition* and the *Condition × Time* interaction reached statistical significance only under the Hotelling–Lawley trace and Roy’s largest root, while Wilks’s λ and Pillai’s trace remained nonsignificant. This suggests that variability in reflectance among exposure conditions was mainly driven by strong effects in a reduced number of response directions. In contrast, no multivariate effects reached significance for **polypropylene**, except for the *Condition × Time* interaction detected via Hotelling–Lawley trace alone. The limited significance observed across multiple statistics indicates that reflectance changes in polypropylene were less structured or exhibited higher intra-group variability. Overall, these results highlight material-dependent differences in how environmental exposure modulates spectral reflectance across time.

### 3.10. General Discussion

The statistical analyses performed in this study provide strong evidence that the evolution of reflectance in automotive plastic components is significantly influenced by the type of polymer, the environmental exposure conditions, and the duration of exposure.

First, three- and two-way ANOVA analyses (Table 6 and Table 12, respectively) show only a significant effect across almost all VIS-bands for Material (excepting Orange and Red) for the material non-specific ANOVA in Table 12. For the material-specific ANOVA summarized in Table 6, no significant effects were detected. These results seem reasonable considering the statistical applicability to the experimental setup under discussion of ANOVA models. Given the multivariate nature of the reflectance data, in which measurements across several spectral bands are inherently correlated, the use of multivariate analysis of variance (MANOVA) is statistically more appropriate than performing multiple separate ANOVAs. Conducting independent ANOVAs for each wavelength or VIS-band increases the risk of inflated type I error rates and fails to capture the underlying covariance structure between dependent variables, as discussed in [59]. MANOVAs address these limitations by testing for group differences across the full vector of dependent variables simultaneously, thereby providing a more comprehensive approach to detecting multivariate effects [60]. In our case, the use of MANOVAs allows for the detection of global degradation patterns in reflectance, which may not be evident when examining each wavelength independently, which seems to be the case with Table 6 results. This strategy is especially valuable when effects are subtle yet consistent across bands, or when interactions affect the multivariate profile rather than individual components. Overall, the application of MANOVA in the present study procures control of statistical error and maximizes the interpretive power of the reflectance dataset by considering both the magnitude and the interdependence of changes across the visible spectrum.

Apropos, the MANOVA models performed in this study (Table 8 and Table 10), the material non-specific MANOVA in Table 8, being a broader scoped analysis than the other, indicated only Material effects, while the one reported in Table 10, broad as well but material-specific to our study case, shows there are indeed discernible effects for Condition, Time, and for Condition × Time in regards to PE, and for Condition and Time for PP. These results sanction us being in the right track to start the general study terms of interest proposed in this paper, with literature antecedents such as [1,3,61,62].

The ANCOVA models (Table 5 and Table 7), which incorporated the initial reflectance as a covariate, demonstrated that baseline differences among samples played a significant role in explaining the observed variability. In the material non-specific analysis shown in Table 5, the covariate was statistically significant in all VIS-bands, suggesting that initial optical characteristics strongly modulate the degradation trajectory. In the same analysis, Condition and Time factors were also significant in most VIS-bands, in complete agreement with what one should expect for environmental exposure. The material specific analysis in Table 7 confirms the previous findings, additionally showing increased prevalence of significant results for PE, which is reasonable considering what has been reported in the literature regarding PE having greater UV exposition susceptibility than PP [57,63,64,65].

In regards to the MANCOVA models (Table 9 and Table 11), material non-specific results in Table 9 portrait significant reflectance effects only through Material × Condition and Material × Condition × Time factors. Material specific results in Table 11 allow us to appreciate significant effects in Condition, Condition × Time for PE, and Condition × Time for PP. These results further reinforce the pattern detected by the previously discussed MANOVA analyses.

**Figure 16:**
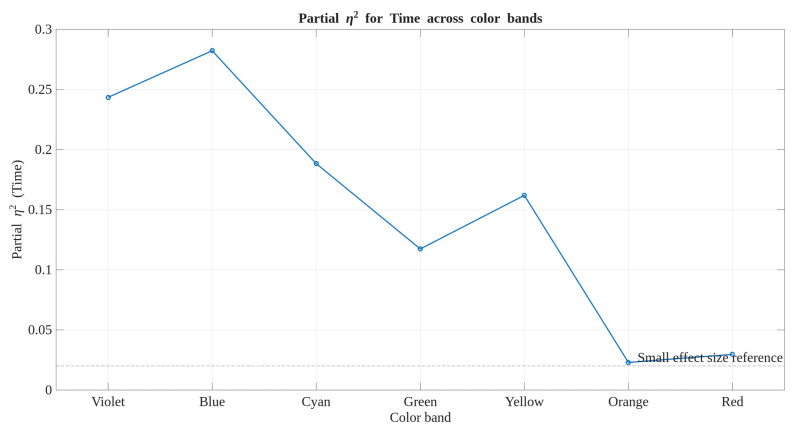
$\eta^{2}$ (*partial effect size*) for the Time factor across VIS-bands. Values were obtained from repeated-measures ANCOVA adjusted for the baseline (T0) reflectance of each band, with *Material* and *Condition* as between-subject factors. The dashed horizontal line marks the threshold for a small effect size ($\eta^{2}=0.02$). Error bars are omitted for clarity.

In addition to the multivariate test statistics performed, $\eta^{2}$ (*partial effect size*) for the *Time* factor using repeated-measures ANCOVA adjusted for the baseline (T0) reflectance of each VIS-band was conducted as well, with *Material* and *Condition* as between-subject factors. The resulting effect-size profile shown in Figure 16 was consistently positive across all bands, indicating that *Time* indeed explained a proportion of variance in reflectance changes regardless of the multivariate statistic contemplated. Effect magnitudes were highest in the Violet and Blue bands ($\eta^{2}\approx 0.24$–0.28), gradually decreasing towards the green and yellow regions, and smallest in the orange and red bands ($\eta^{2}\approx 0.02$–0.03). This gradual spectral pattern supports the interpretation that temporal degradation effects are systematic and wavelength-dependent, even if multivariate *p*-values differ between Pillai’s Trace, Wilks’s Lambda, Hotelling’s Trace, and Roy’s Largest Root. Therefore, the observed variability in statistical significance reflects differences in test sensitivity rather than adversarial/contradictory temporal trends.

**Figure 17:**
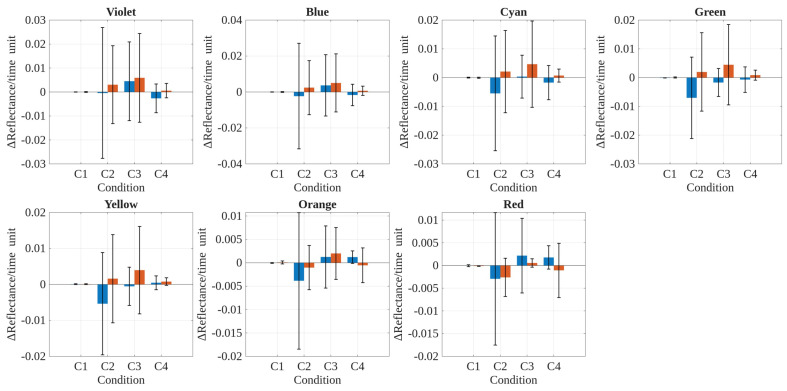
Mean reflectance slopes with respect to time (i.e., ΔReflectance/time unit) for each VIS-band, comparing polyethylene (PE) and polypropylene (PP) under the four exposure conditions (C1–C4). Bars represent the group mean, and error bars indicate the standard deviation across replicates.

On the other hand, Figure 17 shows the variability in slope values (ΔReflectance per time unit) compared between polyethylene (PE) and polypropylene (PP) across the three exposure conditions (C2–C4). In 14 out of the 21 possible cases (7 spectral bands × 3 conditions), the standard deviation for PE exceeded that for PP. This pattern supports the notion that PE generally exhibited greater variability in reflectance change rates under these exposure conditions. The inclusion of both positive and negative slope values in the analysis reflects the actual direction of change in reflectance, rather than only its magnitude. Therefore, this observation supports the interpretation that PE tends to be more sensitive to the tested environmental exposure conditions, whereas PP generally demonstrates greater stability in its optical response over time. This is consistent with established knowledge of polyolefin optical degradation (reflectance changes), since it has been documented that polyethylene is more susceptible to UV exposure optical effects at longer wavelength bands than polypropylene due to the presence of tertiary carbon atoms along its backbone, which serve as preferential sites for radical formation and subsequent chain scission, among other photo-oxidation mechanisms [58]. Again, Figure 17 allows us to appreciate that indeed PE has greater variability in 5 out of 9 occasions (∼60%) than PE for the longer wavelength bands (i.e., Yellow, Orange, and Red). Overall, the present study reinforces the importance of accounting for material-specific degradation pathways and test conditions when designing proper accelerated UV-radiation weathering protocols.

Finally, exposure settings used in this work were designed to modulate the spectral content—chiefly the ultraviolet (UV) component—rather than the intensity of the visible (VIS) band. Polyethylene photodegradation is initiated predominantly by UV photons that generate radicals and carbonyls; by contrast, VIS photons (E<3.1 eV) are typically insufficient to cleave C–C/C–H bonds in the absence of sensitizers. In our data this is reflected by the subject-normalized η(t), which remains ≈0 for the Control (ambient VIS with minimal UV) while increasing under UV-richer conditions. Since VIS intensity was not varied independently, and irradiance was not logged, the present study cannot resolve a dose–response to VIS intensity per se. Future work should decouple spectrum and intensity (e.g., neutral-density attenuation at fixed spectrum, separate UV/VIS channels, and on-site dosimetry) to explicitly test VIS-intensity effects on η(t), as well as accompanying statistical treatment.

### 3.11. Integrated Synthesis of the Results and Discussion

**Overall aim.** A multi-statistical framework was established to quantify optical degradation of polyolefins by combining multivariate descriptors and covariate-adjusted models, and by cross-validating spectral outcomes against mechanical and morphological readouts.
**Data structure and covariates.** The analysis accounts for material (PE, PP), exposure condition (control, glass-protected, unprotected, UV chamber), wavelength/VIS-band, and repeated measurements across indistinguishable specimens (used as a random/covariate factor when appropriate). This enables inference on condition effects while controlling between-specimen variability.
**Multivariate contrasts.** Multivariate comparisons of spectral summaries and colorimetric indices (MANOVA/MANCOVA when covariates apply) consistently separated exposure conditions, with the largest effects under UV chamber and unprotected VIS. Covariate adjustment (e.g., baseline spectral level and specimen) improved power and reduced residual variance relative to unadjusted ANOVA.
**Time-resolved modeling.** For wavelength-resolved and VIS-band-aggregated responses, physically motivated kinetics (first-order, stretched, lag, offset, and bi-exponential) were compared using information criteria (AICc). First-order sufficed at the VIS-band level, while bi-exponential became competitive where early/late processes coexisted. Half-times $t_{1/2}$ and asymptotes could also be extracted to enable condition ranking and cross-material comparisons.
**Spectral change as an efficiency signal.** A bounded, broadband reflectance-based trajectory η(t)∈[0,1] was used as a compact summary of spectral change. η(t) supports the multivariate findings by providing interpretable growth-to-plateau behavior and uncertainty-weighted fits for visualization and meta-comparison.
**Uncertainty and robustness.** Uncertainty via weighted fitting (using within-time dispersion) was reported, considering effect sizes and confidence bounds. Sensitivity analyses (band vs. full-spectrum aggregation, and inclusion/exclusion of lags/offsets) showed stable condition ordering and similar kinetics classes.
**Mechanical consistency (hardness).** Trends in mechanical hardness changes under UV chamber and unprotected VIS paralleled the spectral degradation ranks, reinforcing that the optical pathway captured by the multivariate/covariate-adjusted models is consistent with functional property loss.
**Material-specific notes.** PP typically showed faster early-stage evolution (smaller $t_{1/2}$) than PE under comparable irradiance histories, while PE exhibited clearer late-time plateaus. Glass protection attenuated both the rate and the asymptotic level across materials, consistent with short-λ screening.
**Limitations and scope.** The present study used a nested design in which subjects (Specimen IDs) are uniquely associated with one material (four specimens per material, eight in total). Thus, subjects are *nested* within material rather than crossed across materials. This implies that material effects are estimated from between-subject contrasts within each material, and cannot be disentangled from unobserved subject-level idiosyncrasies beyond those controlled by our normalization. Our subject-normalized spectral efficiency, η(t), reduces between-subject offsets and scaling by (i) using the subject’s own baseline (t=0) within each condition and (ii) scaling by the subject’s baseline spectral norm, thereby emphasizing within-subject temporal change. While this improves comparability and precision for time trends and condition contrasts, it does not change the nesting structure: inference on material differences should be interpreted as conditional on the sampled subjects (four per material) and considered preliminary/exploratory. We therefore report effect sizes and model-selected kinetics (AICc) with appropriate caution, and we refrain from population-wide claims about materials beyond the scope of this nested cohort.

The contribution of this work is then an integrated, covariate-aware, multivariate framework that (i) separates condition effects while controlling specimen heterogeneity, (ii) maps spectral change onto interpretable kinetic summaries for cross-study comparison, and (iii) corroborates optical signatures with mechanical evidence. This synthesis provides a portable analysis blueprint across polyolefin grades, exposure protocols, and instrument settings.

### 3.12. Linking Spectral Trends to Polymer Degradation Mechanisms

The spectral patterns reported here are consistent with established pathways of polyolefin photo-oxidation and weathering. First, the band-wise reflectance decay—more pronounced at shorter VIS wavelengths under UV chamber exposure—agrees with wavelength selectivity of photo-oxidation and the higher quantum efficiency of shorter-λ irradiation [4,10,44]. This selectivity is compatible with early-stage chromophore formation and surface carbonyl build-up that drive increased absorption/scattering and thus reduced reflectance [35,47].

Second, the observed multi-timescale kinetics (cases where a constrained bi-exponential competed with first-order fits) align with concurrent fast surface processes (radical initiation/oxygen uptake) and slower propagation/oxidative embrittlement, as reported for PE/PP under accelerated and natural weathering [49,58]. Depth-sensitive studies have shown near-surface oxidation gradients [57]; such gradients can manifest kinetically as an apparent lag or stretched response in bulk optical signals, consistent with our occasional δ>0 (lag) or β<1 (stretched) selections.

Third, condition-wise contrasts match practical expectations: The UV chamber (UVA/UVB fluorescent lamps) accelerates damage relative to outdoor exposure, while glass shielding attenuates high-energy radiation and slows spectral change [17,21]. Material-wise, PP’s larger hardness losses alongside a more variable reflectance trajectory point to morphology/additive dependencies frequently seen in automotive grades and recycled streams [3].

Overall, the statistical evidence (RM-MANCOVA/ANCOVA and kinetic summaries) provides a mechanistic narrative: *(i)* early, wavelength-sensitive oxidation dominates the visible response; *(ii)* multi-pathway kinetics are plausible in some configurations; and *(iii)* exposure protocol and shielding modulate rates and asymptotes in line with known chemistry of PE/PP photoaging. These connections strengthen the materials-science interpretation of the findings without altering the inferential framework.

## 4. Conclusions

The results of this study demonstrate that visible—VIS—reflectance behavior of automotive polyolefins under UV-radiation stress is strongly influenced by environmental exposure, material type, and time. The reflectance dataset, measured across seven visible VIS-bands and analyzed using ANOVA, MANOVA, ANCOVA, and MANCOVA statistical models, revealed that both PE and PP respond distinctly to UV-radiation weathering/aging conditions. In addition, statistical models conducted revealed that both materials underwent changes in hardness as well, an indicator of accompanying mechanical degradation. In particular, hardness decreased in most exposed groups, with UV chamber and unprotected outdoor exposure showing the greatest reductions.

Generally speaking, PE showed more gradual and consistent reflectance reduction across VIS-bands, whereas PP presented more variable responsiveness, particularly under UV chamber (ASTM G154 protocol) exposure. The repeated-measures MANOVA models identified present main effects for Time and Condition, while the ANCOVA models confirmed the importance of adjusting for initial reflectance to account for its spectral evolution over time, besides highlighting a range of both main or second order material-differentiated effects. MANCOVA results manifested that the interaction between Condition and Time was the most robust predictor of spectral evolution in the dataset. These trends were mutually reinforced by the set of conducted analyses, which validated the statistical robustness of the findings across VIS-bands. These results reinforce the necessity of covariate adjustment in optical degradation/aging studies and spotlight the practical value of using UV chamber tests to emulate short/medium/long-term outdoor effects. The results also align with prior literature documenting the molecular mechanisms of polyolefin degradation, including chain scission and oxidation in both PE and PP automotive components.

Therefore, findings support the use of multivariate and covariate-adjusted statistical frameworks for evaluating optical degradation/aging in polymers, combining approaches to capture both individual and collective spectral behavior, and suggest that PP may be more sensitive to exposure condition variability, while PE exhibits steadier degradation patterns under similar stressors. Additionally, they are relevant in the fields of material selection and accelerated testing protocols design and standardization regarding the automotive industry and governmental regulatory agencies, supporting the use of VIS reflectance analysis as a non-destructive tool for evaluating photo-induced degradation/aging/weathering in polymer components.

### 4.1. Preliminary Implications of the Results

Results presented provide concrete guidance for both materials selection and degradation modeling in automotive applications:

1. **Balance between appearance and mechanical retention.** Across exposure scenarios, PP tended to show *smaller net changes in VIS reflectance* (cosmetic change) to PE while exhibiting a *larger decrease in Shore D hardness*. In practice, PP can be preferable for parts where color/appearance in the visible range dominates acceptance criteria (e.g., trims within the field of view), whereas PE is comparatively safer where mechanical retention under ageing is the primary constraint. Designers should trade off these trends according to part function.
2. **Exposure management and shielding.** The “glass-protected” condition consistently attenuated VIS reflectance change relative to “unprotected” outdoor exposure, and the UV chamber produced faster and larger spectral shifts. For under-glazing components or where coatings/films are feasible, adopting transparent shields or UV-absorbing layers reduces cosmetic drift. When specifying laboratory testing, the chamber protocol is a *conservative* surrogate for outdoor weathering to rank alternatives.
3. **Band-aware monitoring.** Summarising reflectance into seven VIS-bands offers a compact, production-friendly QC signal. Tracking the bands most relevant to brand colour targets helps detect early drift without full spectral processing. The figures show the time course per band (mean ± SEM, scaled for clarity), enabling side-by-side comparison of materials and conditions.
4. **Kinetic reading of the curves.** The fitted exponential family supplies a kinetic lens over spectral trajectories. Although η(t) is reported here *graphically* (with optional half-time $t_{1/2}$ and asymptote $\eta_{\infty }$ discussed but not tabulated), the relative ordering of time scales and plateaus can be inferred from the curves: faster initial changes under chamber exposure along with more coherent (smoother) trajectories for PE and more band-specific responses for PP.
5. **Test and inspection planning.** Given the segmented evolution (earlier drift within the first interval and slower change thereafter), front-loaded inspections (e.g., during early service) are advisable for appearance-critical PP parts, while mechanical-critical PE parts merit periodic hardness checks throughout service life. Chamber-to-field factors can be established by matching the early η(t) rise in the chamber to acceptable outdoor drift.
6. **Modelling pragmatics.** Information-criterion selection consistently favored simple exponentials per VIS-band, with stretched or bi-exponential models only where justified by data. For routine programs we recommend the following: (1) per-band first-order fits as default; (2) AICc-based escalation only if residual structure persists; (3) reporting mean ± SEM and the selected model per condition for transparency.

### 4.2. Limitations and Scope

As stated earlier in this manuscript, these implications are preliminary given the sample size and the material–subject linkage in this dataset. Estimates should be read as patterns and rankings rather than population parameters. Nevertheless, the patterns are internally consistent across bands; conditions and hardness and provide actionable guidance for part selection and qualification testing.

## Figures and Tables

**Table 1:** Features of the Gain Express Holdings Ltd. 560-10D Shore D digital durometer used.

Feature	Description
Sharp cone point	SR 0.1 [mm], 30° including angle.
Measure sample thickness	>6 [mm] (no more than 3 layers).
Measured area	Not less than the pressure edge of 15 [mm].
LCD display	4 digits, 40 × 18 [mm] (1.57×) 0.71 [inch].
Measuring range/Graduation	0–100 HD.
Indentor running	0–2.2 [mm].
Indentor press on telos	A, C type 0.55–8.06 [N], D type 0–44.5 [N].
Resolution	0.5 HD.
Power supply	1 × SR44 button cell battery.
Item size	87 [mm] × 56 [mm] × 25 [mm] (L × W × D) (3.43″ × 2.2″ × 0.98″)
Item weight	36 [g] (4.79 [oz]).

**Table 2:** Features of the Konica Minolta CM-700d spectrophotometer used.

Feature	Description
Illumination/viewing system	Diffused illumination, 8° viewing angle.
	Specular component included/excluded.
	Conforms to CIE No. 15 ISO 7724/1, DIN5033 Teil7,
	ASTM E 1164, and JIS Z 8722.
Size of integrating sphere/diameter	40 [mm].
Spectral separation device	Diffraction grating.
Wavelength range	400 to 700 [nm].
Wavelength pitch	10 [nm].
Half bandwidth	≃10 [nm].
Reflectance range	0 to 175%, display resolution: 0.01%
Light source	Pulsed Xenon Lamp (with UV cut filter).
Measurement time	≃1 [s].
Minimum measurement interval	≃2 [s] (in SCI or SCE mode).

**Table 3:** Representative literature on polyolefin degradation quantification contrasted with the present VIS–reflectance framework. Prior work mostly quantifies damage via chemical or color indices (e.g., carbonyl index, yellowness), gas evolution, or mechanical retention; here, we use band-aggregated visible reflectance tracked over time and visualize a bounded, model-based trajectory η(t) as a compact descriptor of spectral change. Kinetic constants ($t_{1/2}$, $\eta_{\infty }$) are discussed conceptually but are not tabulated in this version. Metrics and models are as reported by each source.

Study	Material(s)	Illumination/Exposure	Metric (Proxy)	Reported Kinetics/ Key Outcome
Gulmine et al. (2003) [35]	PE (LDPE)	Artificial accelerated weathering	FTIR carbonyl index; UV-VIS; morphology	CI increases with dose; qualitative kinetics; links spectral growth to surface oxidation.
Fernando et al. (2007) [49]	PE, PP	Photodegradation (UV)	CO_2_ evolution; CI	Oxidation/gas-evolution correlates with CI; effective first-order-like regimes discussed.
Shyichuk et al. (2005) [57]	PE, PP, E–P copolymer	UV exposure; depth profiles	Depth-resolved CI	Near-surface oxidation dominates; gradients consistent with short-λ absorption, implicit multi-timescale behavior.
Tocháček & Vrátničková (2014) [17]	PP and copolymers	UV accelerated; temperature effects	Mechanical retention; color/CI	Temperature accelerates damage; Arrhenius-type behavior; practical lifetime prediction.
Rodríguez et al. (2020) [8]	LDPE	UV-aging	Tensile/fracture metrics	Monotonic loss of toughness; time-to-threshold comparisons; no explicit spectral efficiency.
Grause et al. (2020) [58]	LDPE, HDPE, PP	Natural/outdoor and accelerated UV weathering (survey across setups)	FTIR, UV-VIS, DSC, mechanical testing	Carbonyl Index (CI), yellowness index, crystallinity increase, tensile retention.
Bourgogne et al. (2022) [44]	Polymers (incl. polyolefins context)	LED weathering, wavelength-resolved	Chemical/CI changes	Shorter wavelengths drive faster photooxidation; wavelength selectivity highlighted.
Du et al. (2024) [21]	PE sheets (types)	UV; varied parameters	Multi-technique spectral/material indices	Factor sensitivity (grade, thickness, stabilizers); qualitative kinetics under parameter sweeps.
Arese et al. (2025) [3]	Recycled PP	UV ageing	Morphological, thermal, mechanical	Stabilization/processing history modulate ageing; no unified bounded spectral metric.
**This work**	PE, PP	VIS: unprotected vs. glass-protected; UV chamber	**Band-aggregated VIS reflectance** (7 bands; full spectrum inspected); η(t)∈[0,1] visualized	**Compact, model-based spectral trajectories** selected by small-sample information criteria; graphical comparison across materials/conditions/bands; kinetic constants discussion.

**Table 4:** Statistical analysis summary of Shore D hardness measurements for polyethylene and polypropylene samples before and after environmental exposure. Paired tests (parametric or nonparametric depending on normality) compared each post-exposure condition to the reference (unexposed, *reference hardness*, i.e., *RH*) state. Complementarily, a one-way ANOVA with Tukey’s post hoc comparisons was conducted separately for each material to assess overall differences across exposure conditions. Each result indicates whether the difference was statistically significant at the α=0.05 level.

Material	Test Type	Results
Polyethylene	Paired comparisons (vs. *RH*)	Outdoor exposure without glass (*OEW/OG*): p<0.05 Outdoor exposure with glass (*OEWG*): p>0.05 Simulated UV chamber (*UVC*): p<0.05
	One-way ANOVA + Tukey	Global test: p<0.05 Significant differences: *RH-OEW/OG*, *RH-UVC*
Polypropylene	Paired comparisons (vs. *RH*)	Outdoor exposure without glass (*OEW/OG*): p<0.05 Outdoor exposure with glass (*OEWG*): p<0.05 Simulated UV chamber (*UVC*): p<0.05
	One-way ANOVA + Tukey	Global test: p>0.05 Significant differences: *RH-OEW/OG*, *RH-OEWG*, *RH-UVC*

**Table 5:** ANCOVA significance summary for mean reflectance in each visible VIS-band. The model includes **Material** (polyethylene or polypropylene), **Condition** (four environmental exposure scenarios), **Time** (T1–T4), and the covariate *initial reflectance* measured at T0. All main effects and interaction terms up to the four-way level were tested. Cells indicate whether each effect was statistically significant (p<0.05) or not (p>0.05). This full-factorial model controls for initial reflectance differences and evaluates the extent to which reflectance changes depend on material type, exposure conditions, and time.

VIS-Band	Material	Condition	Time	Covariate	M × C	M × T	M × Cov	C × T	C × Cov	T × Cov	M × C × T	M × C × Cov	M × T × Cov	C × T × Cov	M × C × T × Cov
Violet	p>0.05	p<0.05	p<0.05	p<0.05	p>0.05	p>0.05	p>0.05	p>0.05	p<0.05	p>0.05	p>0.05	p>0.05	p>0.05	p>0.05	p>0.05
Blue	p<0.05	p<0.05	p<0.05	p<0.05	p<0.05	p>0.05	p>0.05	p>0.05	p<0.05	p<0.05	p>0.05	p>0.05	p>0.05	p>0.05	p>0.05
Cyan	p<0.05	p<0.05	p<0.05	p<0.05	p>0.05	p>0.05	p>0.05	p>0.05	p<0.05	p<0.05	p>0.05	p>0.05	p>0.05	p>0.05	p>0.05
Green	p>0.05	p<0.05	p>0.05	p<0.05	p>0.05	p>0.05	p>0.05	p>0.05	p<0.05	p<0.05	p>0.05	p>0.05	p>0.05	p>0.05	p>0.05
Yellow	p>0.05	p<0.05	p<0.05	p<0.05	p>0.05	p>0.05	p>0.05	p>0.05	p<0.05	p<0.05	p>0.05	p>0.05	p>0.05	p>0.05	p>0.05
Orange	p>0.05	p>0.05	p>0.05	p<0.05	p<0.05	p>0.05	p>0.05	p>0.05	p<0.05	p>0.05	p>0.05	p<0.05	p<0.05	p>0.05	p>0.05
Red	p>0.05	p<0.05	p>0.05	p<0.05	p<0.05	p>0.05	p>0.05	p>0.05	p<0.05	p>0.05	p>0.05	p<0.05	p<0.05	p>0.05	p>0.05

**Table 6:** Two-way ANOVA (*p* values) evaluating the effects of exposure **Condition** (C1: no exposure, C2: exposure under glass, C3: exposure without glass, C4: artificial UV exposure; ASTM G154 protocol), **Time** (T0 to T4), and their interaction on the average visible reflectance of automotive plastic specimens. The response variable is the mean reflectance within each visible VIS-band, calculated as the average reflectance over three adjacent wavelengths for each VIS-band (Violet to Red). The analysis was conducted separately for each material: polyethylene (PE) and polypropylene (PP). Each cell indicates whether the effect was statistically significant at the α=0.05 level.

Color	PE Cond	PE Time	PE Cond × Time	PP Cond	PP Time	PP Cond × Time
Violet	*p* > 0.05	*p* > 0.05	*p* > 0.05	*p* > 0.05	*p* > 0.05	*p* > 0.05
Blue	*p* > 0.05	*p* > 0.05	*p* > 0.05	*p* > 0.05	*p* > 0.05	*p* > 0.05
Cyan	*p* > 0.05	*p* > 0.05	*p* > 0.05	*p* > 0.05	*p* > 0.05	*p* > 0.05
Green	*p* > 0.05	*p* > 0.05	*p* > 0.05	*p* > 0.05	*p* > 0.05	*p* > 0.05
Yellow	*p* > 0.05	*p* > 0.05	*p* > 0.05	*p* > 0.05	*p* > 0.05	*p* > 0.05
Orange	*p* > 0.05	*p* > 0.05	*p* > 0.05	*p* > 0.05	*p* > 0.05	*p* > 0.05
Red	*p* > 0.05	*p* > 0.05	*p* > 0.05	*p* > 0.05	*p* > 0.05	*p* > 0.05

**Table 7:** ANCOVA significance summary for average reflectance in each visible VIS-band, analyzed separately for **polyethylene** and **polypropylene**. Each model included the fixed factors **Condition** (four environmental exposure scenarios) and **Time** (four sampling points: $T_{1}$ to $T_{4}$), as well as the covariate *Initial Reflectance* measured at baseline ($T_{0}$). All second-order interactions (Condition × Time, Condition × Covariate, Time × Covariate) and the third-order interaction (Condition × Time × Covariate) were also tested. The table reports whether each effect was statistically significant (p<0.05) or not (p>0.05), based on individual ANCOVA models fitted for each visible VIS-band.

Material	VIS-Band	Condition	Time	Covariate	C × T	C × Cov	T × Cov	C × T × Cov
Polyethylene	Violet	p<0.05	p>0.05	p<0.05	p>0.05	p<0.05	p<0.05	p>0.05
Polyethylene	Blue	p<0.05	p<0.05	p<0.05	p>0.05	p<0.05	p<0.05	p>0.05
Polyethylene	Cyan	p<0.05	p>0.05	p<0.05	p>0.05	p<0.05	p<0.05	p>0.05
Polyethylene	Green	p<0.05	p>0.05	p<0.05	p>0.05	p<0.05	p>0.05	p>0.05
Polyethylene	Yellow	p<0.05	p>0.05	p<0.05	p>0.05	p<0.05	p<0.05	p<0.05
Polyethylene	Orange	p<0.05	p>0.05	p<0.05	p>0.05	p<0.05	p<0.05	p<0.05
Polyethylene	Red	p<0.05	p>0.05	p<0.05	p>0.05	p<0.05	p<0.05	p>0.05
Polypropylene	Violet	p>0.05	p>0.05	p<0.05	p>0.05	p>0.05	p>0.05	p>0.05
Polypropylene	Blue	p>0.05	p>0.05	p<0.05	p>0.05	p>0.05	p>0.05	p>0.05
Polypropylene	Cyan	p>0.05	p>0.05	p<0.05	p>0.05	p>0.05	p>0.05	p>0.05
Polypropylene	Green	p>0.05	p>0.05	p<0.05	p>0.05	p>0.05	p>0.05	p>0.05
Polypropylene	Yellow	p>0.05	p>0.05	p<0.05	p>0.05	p>0.05	p>0.05	p>0.05
Polypropylene	Orange	p<0.05	p>0.05	p<0.05	p>0.05	p<0.05	p<0.05	p>0.05
Polypropylene	Red	p<0.05	p>0.05	p<0.05	p>0.05	p<0.05	p>0.05	p>0.05

**Table 8:** Multivariate analysis of variance (MANOVA) assessing the effects of **Material** (polyethylene, polypropylene), **Condition** (C1: no exposure; C2: environmental exposure under glass; C3: environmental exposure without protection; C4: UV chamber, ASTM G154 protocol), **Time** (T0 to T4), and their interactions on the multivariate response of average spectral reflectance. The table summarizes the statistical significance of each factor and interaction across four multivariate test statistics: Wilks’s λ, Pillai’s trace, Hotelling–Lawley trace, and Roy’s greatest root.

Effect	Test	Significance
Material	Wilks’s λ	p<0.05
	Pillai’s trace	p<0.05
	Hotelling–Lawley trace	p<0.05
	Roy’s greatest root	p<0.05
Condition	Wilks’s λ	p>0.05
	Pillai’s trace	p>0.05
	Hotelling–Lawley trace	p>0.05
	Roy’s greatest root	p>0.05
Time	Wilks’s λ	p>0.05
	Pillai’s trace	p>0.05
	Hotelling–Lawley trace	p>0.05
	Roy’s greatest root	p>0.05
Material × Condition	Wilks’s λ	p>0.05
	Pillai’s trace	p>0.05
	Hotelling–Lawley trace	p>0.05
	Roy’s greatest root	p>0.05
Material × Time	Wilks’s λ	p>0.05
	Pillai’s trace	p>0.05
	Hotelling–Lawley trace	p>0.05
	Roy’s greatest root	p>0.05
Condition × Time	Wilks’s λ	p>0.05
	Pillai’s trace	p>0.05
	Hotelling–Lawley trace	p>0.05
	Roy’s greatest root	p>0.05
Material × Condition × Time	Wilks’s λ	p>0.05
	Pillai’s trace	p>0.05
	Hotelling–Lawley trace	p>0.05
	Roy’s greatest root	p>0.05

**Table 9:** Multivariate significance summary from a MANCOVA model adjusted by initial reflectance (T0) for each average visible VIS-band. The response matrix includes the average reflectance values for seven spectral bands (Violet, Blue, Cyan, Green, Yellow, Orange, and Red) across 224 observations. The model tests the main effects of **Material** (polyethylene or polypropylene), **Condition** (four levels of environmental exposure), and **Time** (four sampling stages from T1 to T4), as well as second- and third-order interactions between these factors. Each cell indicates whether the corresponding effect was statistically or not according to four multivariate test statistics: Wilks’s λ, Pillai’s trace, Hotelling–Lawley trace, and Roy’s largest root.

Effect	Wilks’s λ	Pillai’s Trace	Hotelling–Lawley	Roy’s Largest Root
Material	p>0.05	p>0.05	p>0.05	p>0.05
Condition	p>0.05	p>0.05	p>0.05	p>0.05
Time	p>0.05	p>0.05	p>0.05	p>0.05
Material × Condition	p>0.05	p>0.05	p<0.05	p>0.05
Material × Time	p>0.05	p>0.05	p>0.05	p>0.05
Condition × Time	p>0.05	p>0.05	p>0.05	p>0.05
Material × Condition × Time	p>0.05	p>0.05	p<0.05	p>0.05

**Table 10:** Multivariate analysis of variance (MANOVA) results for the repeated-measures effects of *Condition*, *Time*, and their interaction on the mean reflectance across seven visible VIS-bands (Violet to Red). The analysis was conducted separately for polyethylene and polypropylene samples. Each entry indicates whether the corresponding multivariate test (Wilks’s Lambda, Pillai’s Trace, Hotelling–Lawley Trace, Roy’s Greatest Root) yielded statistical significance (p<0.05) or not (p>0.05). Both *Condition* and *Time* were treated as within-subjects factors in the repeated-measures design.

Effect	Polyethylene				Polypropylene			
	Wilks	Pillai	Hotelling	Roy	Wilks	Pillai	Hotelling	Roy
Condition	p<0.05	p<0.05	p<0.05	p<0.05	p>0.05	p>0.05	p>0.05	p<0.05
Time	p>0.05	p>0.05	p>0.05	p<0.05	p>0.05	p>0.05	p>0.05	p<0.05
Condition × Time	p>0.05	p>0.05	p>0.05	p<0.05	p>0.05	p>0.05	p>0.05	p>0.05

**Table 11:** Multivariate significance summary from two separate MANCOVA models conducted for polyethylene and polypropylene, respectively. The response matrix consists of the average reflectance values computed for each of the seven visible VIS-bands measured at four post-exposure stages (T1 to T4). Each model includes two fixed between-subject factors: **Condition** (four levels of environmental exposure) and **Time** (T1 to T4), as well as their second-order interaction (**Condition × Time**). The initial reflectance (T0) was included as a covariate to control for baseline variability. Statistical significance (p<0.05 or p>0.05) was evaluated using four classical multivariate test statistics: Wilks’s λ, Pillai’s trace, Hotelling–Lawley trace, and Roy’s largest root.

Material	Effect	Wilks’s λ	Pillai’s Trace	Hotelling–Lawley Trace	Roy’s Largest Root
Polyethylene	Condition	p>0.05	p>0.05	p<0.05	p<0.05
	Time	p>0.05	p>0.05	p>0.05	p>0.05
	Condition × Time	p>0.05	p>0.05	p<0.05	p<0.05
Polypropylene	Condition	p>0.05	p>0.05	p>0.05	p>0.05
	Time	p>0.05	p>0.05	p>0.05	p>0.05
	Condition × Time	p>0.05	p>0.05	p<0.05	p>0.05

**Table 12:** ANOVA results (*p* values) for average reflectance per VIS-band, including the interaction between Material (**M**), Condition (**C**), and Time (**T**). Each cell indicates whether the effect was statistically significant at the α=0.05 level. Summary of significance thresholds (*p* values) from a three-way ANOVA performed on the average reflectance per VIS-band. The response variable is the mean reflectance computed for each band of the visible spectrum (Violet to Red), based on the average of three adjacent wavelengths. The between-subject factors considered are **Material** (polyethylene or polypropylene), **Condition** (four levels of environmental exposure), and **Time** (five measurement times). Each cell indicates whether the corresponding main effect or interaction was statistically significant at α=0.05.

Color	Material	Condition	Time	M × C	M × T	C × T	M × C × T
Violet	p<0.05	p>0.05	p>0.05	p>0.05	p>0.05	p>0.05	p>0.05
Blue	p<0.05	p>0.05	p>0.05	p>0.05	p>0.05	p>0.05	p>0.05
Cian	p<0.05	p>0.05	p>0.05	p>0.05	p>0.05	p>0.05	p>0.05
Green	p<0.05	p>0.05	p>0.05	p>0.05	p>0.05	p>0.05	p>0.05
Yellow	p<0.05	p>0.05	p>0.05	p>0.05	p>0.05	p>0.05	p>0.05
Orange	p>0.05	p>0.05	p>0.05	p>0.05	p>0.05	p>0.05	p>0.05
Red	p>0.05	p>0.05	p>0.05	p>0.05	p>0.05	p>0.05	p>0.05

## Data Availability

The raw data supporting the conclusions of this article will be made available by the authors on request.

## Abbreviations

The following abbreviations are used in this manuscript:

ANOVA	Analysis of Variance
ANCOVA	Analysis of Covariance
MANCOVA	Multivariate Analysis of Covariance
MANOVA	Multivariate Analysis of Variance
PE	Polyethylene
PP	Polypropylene
UV	Ultraviolet
VIS	Visible
